# In vivo proximity proteomics of nascent synapses reveals a novel regulator of cytoskeleton-mediated synaptic maturation

**DOI:** 10.1038/s41467-019-08288-w

**Published:** 2019-01-23

**Authors:** Erin F. Spence, Shataakshi Dube, Akiyoshi Uezu, Margaret Locke, Erik J. Soderblom, Scott H. Soderling

**Affiliations:** 1 Department of Cell Biology, Duke University Medical Center, Durham, NC 27710 USA; 2 Department of Neurobiology, Duke University Medical Center, Durham, NC 27710 USA; 3 Duke Proteomics and Metabolomics Shared Resource, Duke Center for Genomic and Computational Biology, Duke University Medical Center, Durham, NC 27710 USA

## Abstract

Excitatory synapse formation during development involves the complex orchestration of both structural and functional alterations at the postsynapse. However, the molecular mechanisms that underlie excitatory synaptogenesis are only partially resolved, in part because the internal machinery of developing synapses is largely unknown. To address this, we apply a chemicogenetic approach, in vivo biotin identification (iBioID), to discover aspects of the proteome of nascent synapses. This approach uncovered sixty proteins, including a previously uncharacterized protein, CARMIL3, which interacts in vivo with the synaptic cytoskeletal regulator proteins SrGAP3 (or WRP) and actin capping protein. Using new CRISPR-based approaches, we validate that endogenous CARMIL3 is localized to developing synapses where it facilitates the recruitment of capping protein and is required for spine structural maturation and AMPAR recruitment associated with synapse unsilencing. Together these proteomic and functional studies reveal a previously unknown mechanism important for excitatory synapse development in the developing perinatal brain.

## Abstract (web summary)

The internal molecular mechanisms that underlie excitatory synaptogenesis remain poorly characterized. This study utilizes a chemogenetic approach, in vivo biotin identification (iBioID), to discover previously uncharacterized proteins at nascent synapses. CARMIL3 is identified as a cytoskeletal protein that facilitates spine maturation and AMPAR recruitment.

## Introduction

During early postnatal development, excitatory postsynapses of the cortex and hippocampus undergo profound morphological and functional changes as synapses mature, which begins with the emergence of dendritic filopodia^1–5^. In the mid-1990s, imaging studies of CA1 pyramidal neurons in dissociative cultures, as well as hippocampal slices, led to the suggestion that dendritic filopodia are precursors of dendritic spines: filopodia stabilized upon axonal contact, but otherwise remained transient precursors^1,3,4^. The authors of these studies hypothesized that spines are formed by restructuring the actin cytoskeleton of filopodia. Additionally, dendritic filopodia form in response to activity; CA1 pyramidal neurons in hippocampal slice cultures were shown to increase filopodia in response to synaptic stimulation^6^. Moreover, sensory deprivation of animals through whisker trimming led to a 40% reduction in filopodia dynamics as well as an inhibition of cortical map refinement^7^. This suggests that sensory experience can drive synaptic plasticity in dendrites, and dendritic filopodia are a part of that process. In the hippocampus of rat pups, dendritic filopodia rapidly emerge from the dendritic shaft and dynamically explore the surrounding neuropil in search of presynaptic partners^2^. Upon contact with presynaptic terminals, these filopodia are stabilized via rapid calcium transients^8^ and undergo a dramatic morphological transition from thin filopodia to spine structures, which recruit AMPA-type glutamate receptors (AMPARs) in a process termed synapse unsilencing^9^. Driving both the morphological and functional spine maturation is the underlying actin cytoskeleton^5,10,11^. Synaptic actin filaments undergo a sudden structural remodeling from loose linear actin filaments in filopodia to a highly branched network of filaments that are believed to provide force generation for spine head enlargement^12^. Mechanisms that trigger this switch in actin structures remain unknown. Recent evidence demonstrates spine synapses must have an internally guided mechanism for development as their formation can occur independent of glutamate or GABA release^13^. These internal mechanisms remain enigmatic, however, despite clear evidence that abnormalities in spine development are linked to neurodevelopmental disorders such as autism spectrum disorders and intellectual disability^14–17^. Unlike biochemical fractionation methods such as synaptosomal preparations or postsynaptic density fractions to isolate mature excitatory postsynapses, there is currently no way to isolate nascent excitatory synapse compartments of neurons. To do so is a particularly challenging endeavor because developing spines are only transiently present in high numbers for several days postnatally and because there are only a small number of proteins known to localize to and mark dendritic filopodia. The Rac-GAP, Wrp (or srGAP3) is one early synaptogenic protein, which is highly enriched in nascent spines. This targeting of Wrp is solely mediated via an N-terminal phosphoinositide-binding FBAR domain that preferentially localizes to dendritic filopodia^11^.

Here we take advantage of the dendritic filopodial targeting of the WRP-FBAR domain to localize the biotin ligase BirA to developing synapses of the cortex and hippocampus to enable in vivo *Bio*tin *Id*entification (iBioID) of nascent synaptic proteins via proximity labeling. Similar to our work to isolate the proteome of inhibitory postsynapses^18^, this approach allowed the chemicogenetic tagging and subsequent mass spectrometry identification of 60 proteins within nascent dendritic spines. One of the highly enriched proteins was a previously uncharacterized member of a family of actin regulator proteins, CARMIL3.

The CARMIL (*C*apping protein, *Ar*p2/3, *M*yosin*IL*inker) family of proteins was originally identified and described in *A. castellanii* and *Dictyostelium*^19,20^. While there is only one CARMIL family member in amoeba, vertebrates express three proteins, CARMIL1–3. The key function of CARMIL proteins is to deliver actin capping proteins to sites of actin filament remodeling^21^. Indeed, CARMIL1 localizes to lamellipodia (sites of membrane expansion in cells)^21,22^. Depletion of CARMIL1 leads to a loss of lamellipodia in cell cultures^22^, while its overexpression enhances membrane protrusion^21^. Mutation of the CARMIL1 capping protein-binding domain demonstrates the delivery of capping protein to sites of actin remodeling is essential for its effects on lamellipodia^21^. These results are consistent with the essential role of capping protein in facilitating Arp2/3-dependent branched actin filament networks^23–25^.

Using newly developed CRISPR genome editing approaches^26^ we demonstrate that CARMIL3 expression is co-incident with spinogenesis, that it localizes to developing synapses, and that it forms an endogenous complex with Wrp and neuronal actin capping protein. Loss of CARMIL3 impairs the transition from filopodia to spines, synaptic AMPAR surface levels, and AMPAR-mediated postsynaptic currents. Thus, CARMIL3 is a previously unknown factor present in nascent synapses that organizes an actin restructuring complex required to transition filopodia to functional dendritic spines.

## Results

### Wrp-BirA biotinylates proteins within developing spines

**Fig. 1:**
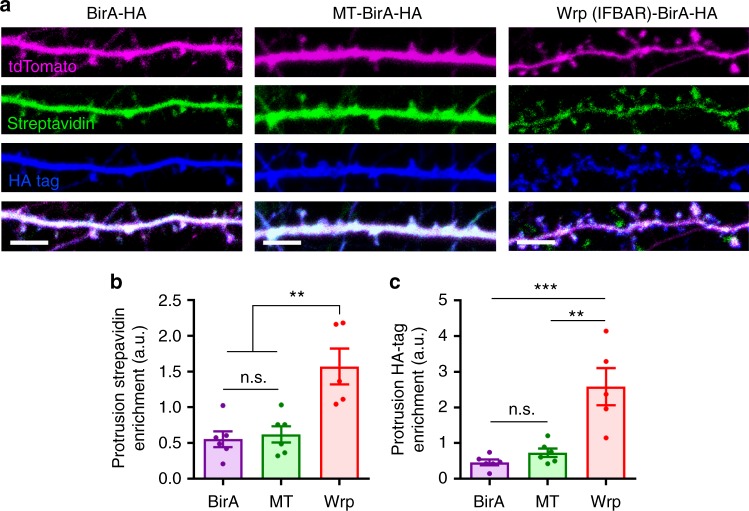
Wrp-BirA localizes to and biotinylates proteins within nascent dendritic spines. **a** Representative images of cultured neurons expressing either BirA (Control), Membrane-Tag-BirA (MT-BirA), or Wrp(IFBAR)-BirA (Wrp-BirA). Co-transfected tandem dimer Tomato fluorescent protein (tdTomato^67^) fill is shown in magenta, streptavidin staining is shown in green, and the HA tag for each construct is shown in blue. Scale bars are 5 μm. **b** Graph depicting streptavidin enrichment in dendritic protrusions vs the dendrite for each BirA (0.555 ± 0.110 spine intensity: dendrite intensity, *n*=6 neurons), MT-BirA (0.622 ± 0.113 spine intensity: dendrite intensity, *n*=6 neurons), and Wrp-BirA (1.353 ± 0.300 spine intensity: dendrite intensity, *n*=6 neurons). *F*_2,15_=5.138, *p*=0.020. **c** Graph depicting streptavidin enrichment in dendritic protrusions vs the dendrite for BirA (0.465 ± 0.080 spine intensity: dendrite intensity, *n*=6 neurons), MT-BirA (0.732 ± 0.118 spine intensity: dendrite intensity, *n*=6 neurons), and Wrp-BirA (2.269 ± 0.530 spine intensity: dendrite intensity, *n*=5 neurons). *F*_2,8.591_=6.452, *p*=0.019. Error bars are standard error of the mean (SEM). *p<0.05; **p<0.01, one-way ANOVAs

Our previous work demonstrated that the Rac-GAP Wrp localizes to dendritic filopodia via the membrane phosphoinositide-binding FBAR domain of Wrp^11^. We found that this domain alone, when expressed in developing neurons, was sufficient to localize GFP to filopodia as well as early developing spines. Recently we utilized fusions between inhibitory synaptic proteins with the promiscuous biotin ligase, BirA (which is similar in size to GFP), to identify proteomes enriched at GABAergic postsynapses by proximity biotinylation in vivo^18^. We thus hypothesized a Wrp-FBAR domain fusion to BirA may enable the localized covalent tagging of the proteome of developing dendritic synaptic structures, which previously have not been amenable to biochemical purification by traditional methods. We tested this possibility by electroporating hippocampal neurons from postnatal day 0 (P0) mice with a tdTomato fill and constructs expressing either BirA alone, Membrane-tagged-BirA (MT-BirA) created from the membrane-targeting region of Gap43, or Wrp-FBAR-BirA (Wrp-BirA). At DIV8 neurons were incubated with biotin overnight, fixed, and stained for tdTomato (αRFP, red), biotinylation (streptavidin, green), and BirA, (αHA, blue) (Fig. 1a). The enrichment of BirA (HA immunoreactivity) and biotinylated proteins (streptavidin staining) in dendritic protrusions was quantitatively analyzed, demonstrating Wrp-BirA significantly biotinylated proteins in early developing dendritic spines compared to BirA alone or MT-BirA (Fig. 1b, c). Thus, Wrp-BirA may allow for the unbiased labeling and identification of proteins involved in early synapse formation.

### Identification of proteome composition of nascent synapses

**Fig. 2:**
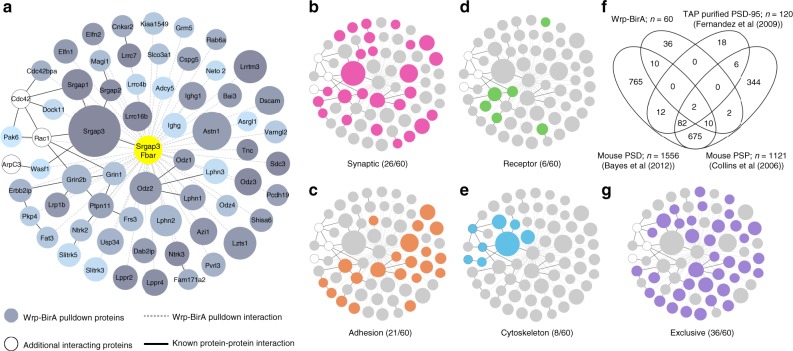
Proteome of developing dendritic protrusions. **a** Wrp-BirA-dependent iBioID identified a network of known and unknown proteins enriched in dendritic filopodia early in spinogenesis. Node titles correspond to the gene name, size represents fold enrichment over the BirA negative control (range 2.45–23.73), shading represents *p*-value with light gray being a lower *p*-value and darker gray a higher *p*-value (range 9.06 × 10^−5^–0.05). Edges are marked according to type of interaction. Dashed lines are iBioID interactions where as solid lines are previously known interactions identified in the Genemania, Biogrid, or String databases. **b** Clustergram of synaptic proteins (pink) as identified through DAVID analysis or the G2C database of Cortex PSD Consensus proteins^38^. **c** Clustergram of adhesion proteins (orange) as identified through DAVID analysis. **d** Clustergram of receptor proteins (green) as identified through DAVID analysis. **e** Clustergram of cytoskeletal proteins (blue) as identified through DAVID analysis. **f** Venn diagram of Wrp-BirA proteome compared to three previously published proteomes of mature postsynapses. **g** Clustergram of proteins found exclusively in the Wrp-BirA network in comparison to mature postsynaptic proteomes (purple)

With this new toolset of BirA probes, we next set out to isolate the proteome of developing synapses by modifying the iBioID approach^18^ for early postnatal proximity biotinylation. We injected pregnant females subcutaneously with 5 mM biotin starting at embryonic day 17.5 (E17.5). When mouse pups were born, they were injected with purified AAVs for each iBioID probe. From the time of AAV injection until postnatal day 5 (P5), both mouse pups as well as mothers received daily subcutaneous injections of biotin to ensure robust in vivo biotinylation. Brain samples were collected at P5, a time point in which synaptogenesis is still in the very early stages and filopodia make up about 20% of synaptic contacts^2^. Samples were processed as described previously^18^ and analyzed using label free quantitative high-mass accuracy LC/MS/MS (Supplementary Figure 1a,b). Based on peptide identity, a total of 2490 proteins were identified in all three iBioID samples, which were further analyzed for proteins specific to either the Wrp-BirA or MT-BirA fractions (Supplementary Figure 1c–g). The resulting proteomes specific for each probe consisted of 60 Wrp-BirA (Fig. 2a) and 117 MT-BirA (Supplementary Figure 2a) enriched proteins. These proteomes (Wrp-BirA vs MT-BirA) were distinct with limited overlap (37%), consistent with the membrane localization of each probe but the early developing spine enrichment specific for the Wrp-BirA probe.

Bioinformatic analysis of each proteome (Fig. 2b–e; Supplementary Figure 2b–e) revealed that the Wrp-BirA proteome represents a nexus of proteins implicated in excitatory synaptic function and development. First, the Wrp-BirA network, when compared to the MT-BirA network, was selectivity enriched for Grin2b, which is preferentially recruited to developing spines compared to later in development, when Grin2a is also recruited to dendritic spines^27,28^. The Wrp-BirA isolated complex also specifically contained a large number of adhesion and receptor molecules known to play critical roles in excitatory postsynaptic development, including latrophilins (*Lphn1–3*)^29,30^, *Lrrtm3* (ref. ^31^), *MAGI1* (ref. ^32^), *Dscam* (ref. ^33^), *Bai3* (ref. ^34^), and *Slitrk5* (ref. ^35^) (Fig. 2c, d). Consistent with the central role of the actin cytoskeleton in dendritic spine development, multiple proteins functionally implicated in modulating cytoskeletal dynamics during spinogenesis were identified only in the Wrp-BirA fraction. These included WAVE1 (*Wasf1*), an activator of the Actin Related Protein 2/3 (Arp2/3) complex that functions downstream of Rac signaling (Fig. 2e). All three of these (WAVE1, Arp2/3, and Rac) are critical for generating highly branched actin filaments required for excitatory synapse maturation^5,10,11,36^. Moreover, the finding of WAVE1 enriched in the Wrp-BirA fraction was consistent with our prior work demonstrating the C-terminal SH3 domain of Wrp binds to WAVE1 to form a complex in neurons important for dendritic spine formation and synaptic plasticity^11^. Indeed, we also found peptides not included in the Wrp-BirA bait corresponding to endogenous Wrp (also known as *Srgap3*) and other members of the SrGAP family (*Srgap1 and 2*), highlighting the roles of these endogenous SrGAP family members during early synapse development (Fig. 2a).

Finally, we also analyzed the distribution of 1951 proteins between the Wrp-BirA-enriched proteins and three comprehensive datasets of mature postsynaptic proteomes available on the genes2cognition online database of synaptic proteomes (Fig. 2f). These datasets include TAP affinity purification of PSD-95-associated complexes from transgenic mice;^37^ purified mouse cortical PSDs;^38^ and an extensive postsynaptic proteome (PSP) dataset combining mass spectrometry data from seven PSD experiments, AMPA and NMDA receptor immunopurifications, and NR2B peptide-affinity purification^39^. We found that 36 of our 60 proteins (Fig. 2g) are not found in any of these prior datasets from mature synapses, indicating an enrichment of the Wrp-BirA proteome for early synapse formation. Bioinformatic analysis using DAVID^40^ of these 36 proteins found a significant enrichment for biological processes of “positive regulation of synapse assembly” (*p* = 3.1 × 10^−8^) and “heterophilic cell-cell membrane adhesion molecules” (*p* = 3 × 10^−11^), further supporting the notion that these proteins are involved in synapse formation and maturation.

### Validating proteomic screen candidates

**Fig. 3:**
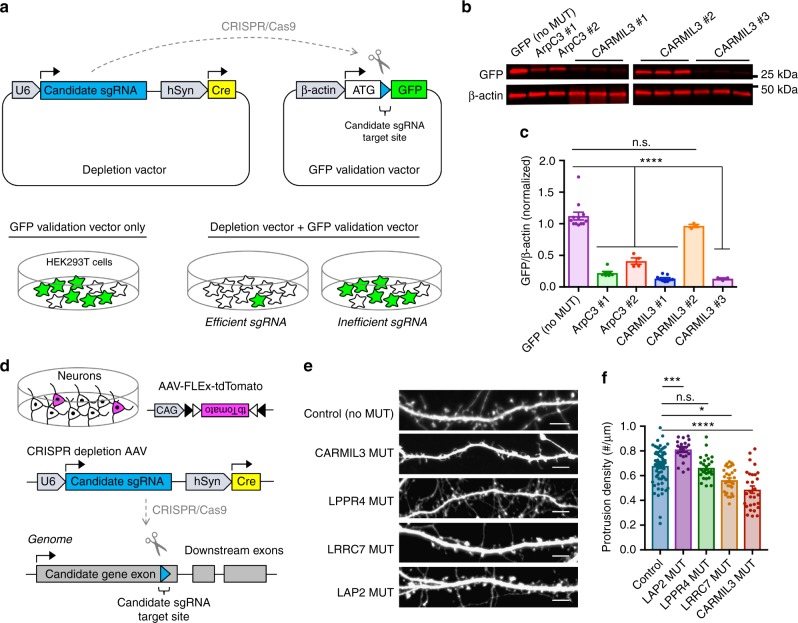
Candidate screening for functional role in filopodia maturation. **a** Schematic depicting the depletion and GFP validation vectors for in vitro guide screening. U6 U6 promoter, sgRNA single guide RNA, hSyn human Synapsin I promoter, Cre cre recombinase, β-actin beta actin promoter, GFP green fluorescent protein. **b** Representative blot for GFP to validate depletion efficiency of sgRNA sequences. **c** Graphical representation of GFP/β-actin intensity relative to no depletion control (GFP alone) (1.12 ± 0.06 GFP intensity, *n*=12 HEK cell samples), ArpC3 #1 (0.22 ± 0.02, *n*=7 samples), ArpC3 #2 (0.41 ± 0.05, *n*=4 samples), CARMIL3 #1 (0.13 ± 0.02, *n*=9 samples), CARMIL3 #2 (0.97 ± 0.02, *n*=3 samples), CARMIL3 #3 (0.10 ± 0.01, *n*=3 samples), LPPR4 #3 (0.04 ± 0.00, *n*=6 samples), LRRC7 #3 (0.06 ± 0.01, *n*=3 samples), and LAP2 #1 (0.02 ± 0.01, *n*=3 samples). *F*_8,41_=85.192, *p*<0.0001. **d** Schematic depicting CRISPR depletion of candidates in cultured hippocampal neurons. **e** Representative images of dendritic morphology for each group of virally mediated depletions. Scale bars are 5 μm. **f** Graphical representation of dendritic protrusion density for each group of virally mediated depletions, Control (66 ± 2 protrusions, *n*=66 neurons), LAP2 (81 ± 2 protrusions, *n*=22 neurons), LPPR4 (66 ± 2 protrusions, *n*=27 neurons), LRRC7 (56 ± 2 protrusions, *n*=30 neurons), CARMIL3 (49 ± 3 protrusions, *n*=29 neurons). Error bars are standard error of the mean (SEM). *F*_4,169_=24.55, *p*<0.0001. *p<0.05, ***p<0.001; ****p<0.0001, one-way ANOVAs

We next tested whether the proteins we identified at developing synapses were functionally required for aspects of spinogenesis in a CRISPR-based imaging screen for dendritic spine formation in vitro. We chose to target the genes encoding the adaptor proteins Erbin (*Lap2)* and Leucine-rich repeat-containing protein 7 (*Lrrc7*, also known as Densin-180), the Lipid Phospholipid Phosphatase-Related Protein Type 4 (*Lppr4*, also known as Plasticity Related Gene Protein 1), and the actin cytoskeletal regulatory capping protein, Arp2/3 and Myosin-I Linker Protein 3 (*Lrrc16b* or *Carmil3*) for CRISPR-based depletion based on insertion–deletion (Indel) mutagenesis. Single guide RNAs (sgRNAs) for each candidate were screened in a cell-based assay that we developed to verify sgRNA efficacy against genomic target sequences cloned in-frame with GFP (GFP validation vector) (Fig. 3a). In this assay, highly efficient sgRNAs are selected based on their ability to reduce the expression of GFP when the genomic sequence they target is placed in-frame following the start methionine.

We first validated this system for identifying functional sgRNAs that alter synaptogenesis by targeting a positive control gene, *ArpC3*, which we previously demonstrated by Cre-*Lox* deletion to significantly alter spine formation^5^. The GFP cell-based assay indicated that the ArpC3 sgRNAs 1 and 2 were both efficacious in mediating Indel formation at their respective genomic target sequences (Fig. 3b, c). Viral expression of Cre and ArpC3 sgRNA 1 in primary hippocampal neurons from *Lox-stop-Lox*-Cas9-P2A-GFP (Cas9 KI) mice phenocopied the increased density of the dendritic filopodial phenotype that we previously reported following the genetic deletion of *ArpC3* (Supplementary Figure 3)^5^. Thus, cell-based GFP-validated sgRNAs can effectively recapitulate morphological alterations in excitatory synapse development downstream of gene disruption.

Using this guide validation approach, three sgRNAs for each candidate of interest were screened. We then picked the most efficient sgRNAs (Fig. 3b, c; Supplementary Figure 4) for viral-mediated expression in cultured hippocampal neurons beginning at DIV0-1 (Fig. 3d). Cultured neurons were fixed at DIV14–16 and immunostained for both GFP (a marker of Cas9 expression) as well as RFP (FLEx-tdTomato; a soluble fill in response to Cre activity). Imaging and analysis of the density and morphology of dendritic protrusions (spines and filopodia) in sgRNA vs negative control (empty sgRNA) was utilized to identify candidates critical for structural aspects of excitatory postsynapse development. Of the four candidates, *Lap2, Lrrc7*, and *Carmil3* each showed significant alterations in the density of dendritic protrusions when compared to negative controls, suggesting they were functionally important for aspects of spinogenesis (Fig. 3e, f). Thus, the results of this functional screen supported the notion that the in vivo proteomics analysis of nascent synapses could successfully identify proteins likely to be critical for aspects of dendritic spine formation and maturation.

### Expression of CARMIL3 during synapse development

**Fig. 4:**
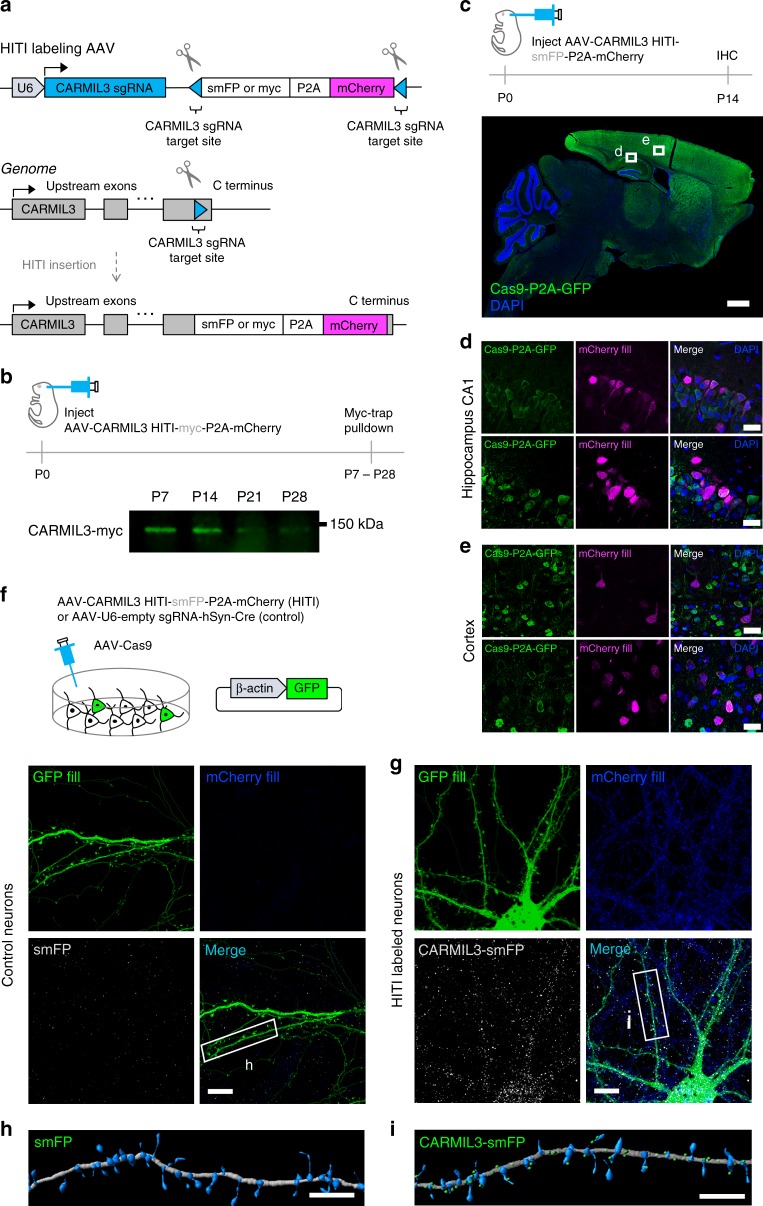
CARMIL3 localization during synapse development. **a** Schematic depicting HITI labeling of endogenous CARMIL3 with myc or smFP. **b** Representative western blot of myc pulldown of endogenous CARMIL3 at P7, P14, P21, and P28. **c** Expression of endogenous CARMIL3 in the P14 mouse brain. CARMIL3 expression is most pronounced in the cortex (green—Cas9-2A-GFP, blue—DAPI). Scale bar is 900 μm. **d** Representative images of HITI-mediated mCherry (magenta) expression in the CA1 hippocampus. Scale bars are 25 μm. **e** Representative images of HITI-mediated mCherry (magenta) expression in the cortex. Scale bars are 25 μm. **f** Control image of AAV-HITI-smFP-P2A-mCherry expression in cultured hippocampal neurons without CARMIL3 sgRNA. Neurons were co-transfected with a GFP fluorescent fill (green) and infected with AAV-Cas9. Scale bar is 5 μm. **g** Knock-in expression of CARMIL3-smFP (gray) in cultured neurons using CARMIL3 sgRNA. CARMIL3 localizes along dendrites and dendritic protrusions in hippocampal neurons and neurons are marked with mCherry expression (blue) under the control of the endogenous CARMIL3 promoter. Neurons were also co-transfected with a GFP fill (green) and infected with AAV-Cas9. Scale bar is 5 μm. **h** IMARIS reconstruction of a dendritic section of a control hippocampal neuron. Dendrite is gray, protrusions are blue, and smFP is green. Scale bar is 5 μm. **i** IMARIS reconstruction of dendritic section of a CARMIL3-smFP KI neuron. Dendrite is gray, protrusions are blue, and CARMIL3-smFP is green. Scale bar is 5 μm

Of the candidates functionally tested above, CARMIL3 depletion led to the greatest phenotype, with a 28% reduction in dendritic protrusions compared to negative controls. CARMIL3 was especially interesting in this regard due to its potential role in regulating actin dynamics as predicted by prior studies of its close homologs, CARMIL1 and CARMIL2^21,22,41^. However, unlike CARMIL1 and 2, the roles of CARMIL3 in actin modulation have not been studied and virtually nothing is known about its potential roles in the developing nervous system. To obviate issues with uncharacterized antibodies, we devised a strategy to tag endogenous CARMIL3 with highly antigenic epitopes detected by well characterized monoclonal antibodies (myc for immunoprecipitation or spaghetti-monster (smFP) that contains 12 HA epitopes for immunolocalization^42^) using Homology-Independent Targeted Integration (HITI)^26^. AAV-HITI vectors with a myc or smFP tag followed by a P2A and mCherry fluorescent protein cassette were designed. These vectors enabled the antigenic tagging of the c-terminus of endogenous CARMIL3 protein while simultaneously marking those neurons with a red fluorescent fill under the endogenous CARMIL3 promoter (Fig. 4a).

We first characterized the expression of CARMIL3 within the cortex and hippocampus during the period of dendritic spine development. P0 Cas9 KI mice were intracortically infused with AAV-CARMIL3 HITI-myc-P2A-mCherry and brain samples were collected at P7, P14, P21, and P28 for immunoprecipitation using a Myc-trap resin, followed by western blot using anti-myc antibodies. CARMIL3 expression was readily detected at P7, but expression declined by P21 and was low to undetectable by P28 (Fig. 4b). These findings supported the initial proteomic data indicating CARMIL3 is expressed during early synapse development.

Using a similar HITI strategy, the regional expression of CARMIL3 within the cortex and hippocampus was also examined. P0 Cas9 KI mice were injected intracortically with AAV-CARMIL3 HITI-smFP-P2A-mCherry, and mice were perfused at P14. The brains were then cryosectioned and immunostained for mCherry as a proxy of CARMIL3 expression (Fig. 4c). mCherry expression was present in pyramidal neurons throughout the cortex and hippocampus, while there was no mCherry immunoreactivity in negative control samples, confirming CARMIL3 expression in these brain regions (Fig. 4d, e; Supplementary Figure 5). Next, the localization of CARMIL3 within hippocampal neurons was assessed based on smFP immunoreactivity. DIV0 cultured mouse hippocampal neurons were sparsely transfected with GFP to visualize morphology followed by infection with AAV-Cas9 and AAV-CARMIL3 HITI-smFP-P2A-mCherry or negative control AAV. Neurons were cultured for 14 days to allow for integration and expression of the smFP cassette at which point neurons were fixed, immunostained for mCherry and smFP, and imaged to localize endogenous CARMIL3. Compared to negative control neurons (Fig. 4f, h), CARMIL3 localized as discrete puncta within dendritic shafts, filopodia, and spines (Fig. 4g, i). Other candidates we identified (Lrrc7 and Lppr4) were also localized in cultured hippocampal neurons using HITI to c-terminal tag the endogenous proteins for immunocytochemistry. Co-localization with the postsynaptic marker Homer1 demonstrated these candidates were also present at synapses (Supplementary Figure 6). Together these data support the localization of several candidates we identified in the Wrp-BirA screen as synaptic proteins. Importantly, endogenous CARMIL3 is also expressed in a temporal and spatial manner consistent with the possibility it facilitates aspects of dendritic spine development.

### Depletion of CARMIL3 reduces dendritic spine density

**Fig. 5:**
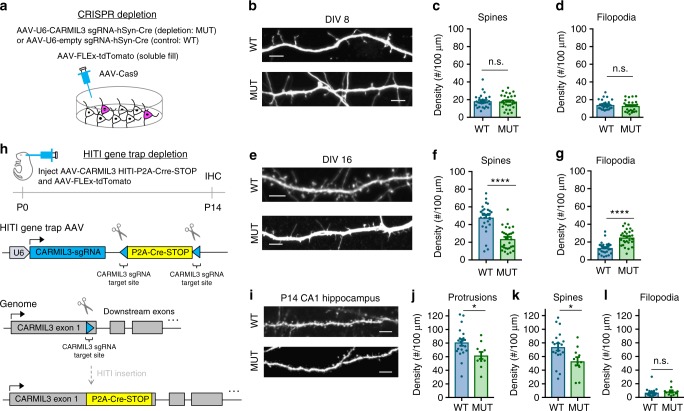
CARMIL3 depletion causes alterations in dendritic spine maturation. **a** Schematic of CRISPR-based depletion strategy of CARMIL3 in cultured hippocampal neurons. **b** Representative images of control (WT) and CARMIL3-depleted (MUT) neurons at DIV8. Scale bars are 5 μm. **c** Graphical representation of dendritic spine density for WT (18 ± 1 spines, *n*=28 neurons) and MUT (18 ± 2 spines, *n*=28 neurons) neurons at DIV8. *p*=0.8235. **d** Graphical representation of filopodia density for WT (14 ± 1 filopodia, *n*=28 neurons) and MUT (13 ± 1 protrusions, *n*=28 neurons) neurons at DIV8. *p*=0.4541. **e** Representative images of WT and MUT neurons at DIV16. Scale bars are 5 μm. **f** Graphical representation of dendritic spine density for WT (48 ± 2 spines, *n*=29 neurons) and MUT (24 ± 2 spines, *n*=29 neurons) neurons at DIV16. *p*<0.0001. **g** Graphical representation of dendritic filopodia density for WT (13 ± 1 filopodia, *n*=29 neurons) and MUT (25 ± 2 filopodia, *n*=29 neurons) neurons at DIV16. *p*<0.0001. **h** Schematic of HITI gene trap strategy utilized for depleting CARMIL3 in vivo. **i** Representative images of tdTomato fill in WT and MUT pyramidal neurons at postnatal day 14 (P14) in hippocampal CA1. Scale bars are 5 μm. **j** Graphical representation of dendritic protrusion density for WT (81 ± 5 protrusions, *n*=20 neurons) and MUT (62 ± 5 protrusions, *n*=11 neurons) neurons at P14. *p*=0.018. **k** Graphical representation of dendritic spine density for WT (74 ± 5 spines, *n*=20 neurons) and MUT (53 ± 6 spines, *n*=11 neurons) neurons at P14. *p*=0.018. **l** Graphical representation of dendritic filopodia density for WT (7 ± 1 filopodia, *n*=20 neurons) and MUT (9 ± 2 filopodia, *n*=11 neurons) neurons at P14. Error bars are standard error of the mean (SEM). *p*=0.272. *p<0.05, **p<0.01, ****p<0.0001, *t*-tests

Next the functional roles of CARMIL3 during spinogenesis were explored using CRISPR-mediated depletion strategies in vitro and in vivo. Based on the initial depletion screening data (Fig. 3f), we further characterized how loss of CARMIL3 alters the morphology of developing synapses at DIV8 and DIV16 in vitro. First, hippocampal neuron cultures from P0 Cas9 KI mice were prepared and infected with AAV-FLEx-tdTomato and either a negative control virus (AAV-empty sgRNA-Cre) or the CARMIL3 sgRNA virus to mutate the gene (MUT) by Indel formation and thus deplete CARMIL3 (Fig. 5a). CARMIL3 sgRNA virus had no effect on the density of dendritic filopodia or spines at DIV8 when compared to negative control treated neurons, suggesting either CARMIL3 is not required for the initial formation of these structures or that the depletion strategy at this early time point was ineffective (Fig. 5b–d). However, at DIV16 (Fig. 5e), a time point when the majority of developing excitatory postsynaptic structures are spines, there was a significant shift in the morphology of CARMIL3-depleted (MUT) neurons compared with control (WT) neurons. The density of dendritic spines was decreased and there were more filopodia-like protrusions in CARMIL3-depleted neurons (Fig. 5f, g). In contrast, mutation of CARMIL3 after the first week of synaptogenesis at DIV10 did not alter spine density at DIV16, suggesting that CARMIL3 may be important for the transition period between filopodia to spines (Supplementary Figure 7c–f). We note, however, that it is also possible the depletion strategy we employed is less effective between DIV10 and 16. To control for possible sgRNA off-target effects, we repeated the analysis of DIV0–16 depletion with a second independent sgRNA against CARMIL3. Neurons treated with this guide also displayed a reduced density of dendritic spines, independently confirming CARMIL3 facilitates spine development (Supplementary Figure 7a, b).

We next tested whether the requirement of CARMIL3 for the development of normal spine density in vitro was also true in vivo. To test this, we intracortically infused P0 pups constitutively expressing Cas9 with AAV-FLEx-tdTomato and a novel HITI Gene Trap virus (Fig. 5h). AAV-HITI-P2A-Cre-STOP inserts a disruptive in-frame P2A sequence followed by Cre, allowing expression of Cre as a sensitive marker of neurons in which the CARMIL3 gene has been interrupted. Negative control virus, AAV-CreERT2, was co-injected with AAV-FLEx-tdTomato at P0 to sparsely label wild-type neurons in vivo following a single intraperitoneal tamoxifen (75 mg/kg) treatment at P7. Coronal brain sections were prepared from P14 mice and the dendritic spines were imaged for changes in density from CA1 apical dendrites (Fig. 5i). Quantitative comparisons between control and CARMIL3 gene-trapped neurons demonstrated the depletion of CARMIL3 in vivo also leads to a significant 28% reduction in dendritic spines (Fig. 5j–l). Together, these data show that CARMIL3 is critical for the development of dendritic spines both in vitro and in vivo.

### Depletion of CARMIL3 alters excitatory synaptic transmission

**Fig. 6:**
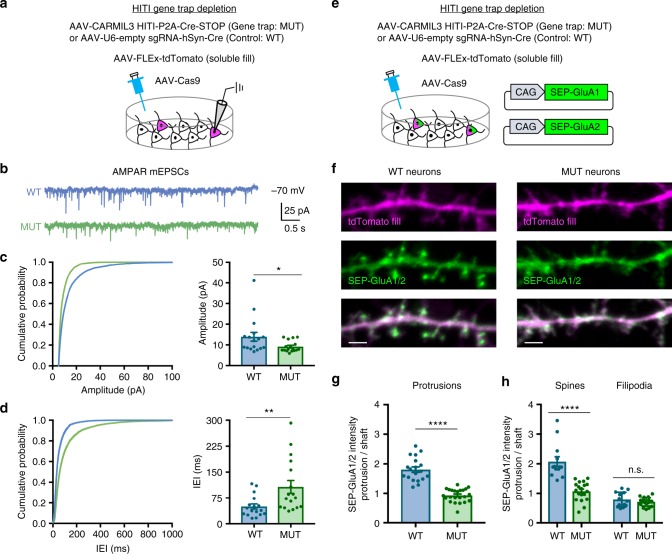
CARMIL3 depletion results in functionally immature excitatory synapses. **a** Schematic of HITI gene trap method used to sparsely deplete CARMIL3 from cultured hippocampal neurons. Whole-cell patch clamp recordings were conducted from tdTomato-expressing cells on DIV12–16. **b** Representative traces of pharmacologically isolated AMPAR-mediated mEPSCs recorded from control (WT, blue) and CARMIL3-depleted (MUT, green) neurons. **c** Amplitude cumulative probability plots for AMPAR-mEPSCs. Quantification on the right shows average AMPAR-mEPSC amplitude for WT (13.92 ± 2.13 pA, *n*=17 neurons) and MUT (9.16 ± 0.64 pA, *n*=17 neurons) neurons. *p*=0.0454. **d** Interevent interval (IEI) cumulative probability plots for AMPAR-mEPSCs. Quantification on the right shows average AMPAR-mEPSC IEI for WT (49.96 ± 7.50 ms, *n*=17 neurons) and MUT (107.22 ± 18.62 ms, *n*=17 neurons) neurons. *p*=0.0095. **e** Schematic of method used to examine surface AMPAR levels in control and CARMIL3-depleted neurons. **f** Representative images of WT and MUT neurons at DIV12–16. tdTomato fill is magenta, and SEP-GluA1/2 is green. Scale bars are 2 μm. **g** Graphical representation of the ratio of SEP-GluA1/2 intensity in protrusions to dendritic shafts for WT (1.81 ± 0.16, *n*=19 neurons) and MUT (0.93 ± 0.05, *n*=23 neurons) neurons. *p* < 0.0001. **h** Graphical representation of the ratio of SEP-GluA1/2 intensity in protrusions to dendritic shafts specifically in spines (WT, 2.07 ± 0.59, *n*=19 neurons; MUT, 1.07 ± 0.32, *n*=23 neurons; *p* < 0.0001) and filipodia (WT, 0.79 ± 0.25, *n* = 19 neurons; MUT, 0.71 ± 0.14, *n* = 23 neurons; *p*=0.319). Error bars are standard error of the mean (SEM). *p < 0.05, **p < 0.01, ****p < 0.0001, *t*-tests

Given the localization and necessity of CARMIL3 for spine development, we next tested whether disruption of CARMIL3 would lead to functional alterations in excitatory synaptic transmission. Hippocampal neurons were co-infected with three AAVs, AAV-Cas9, AAV-FLEx-tdTomato, and gene trap virus (AAV-CARMIL3 HITI-P2A-Cre-STOP) or negative control virus (AAV-empty sgRNA-Cre) (Fig. 6a). This strategy resulted in sparse labeling of CARMIL3 gene-trapped neurons vs matched control neurons without depletion of CARMIL3, both expressing tdTomato. On DIV12–16, somatic whole-cell recordings from tdTomato-expressing neurons were used to pharmacologically isolate AMPA or NMDA receptor-mediated miniature excitatory postsynaptic currents (AMPAR-mEPSCs or NMDAR-mEPSCs) and miniature inhibitory postsynaptic currents (mIPSCs). Compared to control neurons, CARMIL3 gene-trapped neurons had a significant decrease in AMPAR-mEPSC amplitude (34%) and frequency (50%), measured as an increase in interevent interval (Fig. 6b–d). In contrast, there was no significant effect of CARMIL3 disruption on NMDAR-mEPSCs (Supplementary Figure 8a–c). There was also no effect on mIPSCs (Supplementary Figure 8d–f), suggesting that CARMIL3 does not play a role at inhibitory synapses. Finally, CARMIL3 depletion did not alter the kinetics (rise or decay times) of AMPAR-mEPSCs, NMDAR-mEPSCs, or mIPSCs (Supplementary Figure 8g–r), suggesting there was no effect on the subunit composition of glutamate or GABA receptors present at synapses from CARMIL3-depleted neurons.

Because of the specific defect on AMPAR-mediated currents, we next analyzed the surface levels of AMPAR subunits at synapses. Cultured neurons were electroporated with GluA1 and GluA2 tagged with the pH-sensitive GFP variant supereclipitic pHlourin (SEP^43^) and then gene trapped as described above (Fig. 6e). Live imaging was conducted on DIV12–16 for SEP and tdTomato fluorescence (Fig. 6f). There was a 51% reduction in the GluA1–GluA2 surface enrichment within dendritic protrusions from gene-trapped neurons compared with WT neurons (Fig. 6g). This difference was driven by a reduction in surface enrichment specifically in dendritic spines, as filopodia in both groups had similarly low levels of enrichment (Fig. 6h).

Together, these experiments demonstrate CARMIL3 is specifically required for AMPAR-dependent excitatory synaptic transmission, but not for inhibitory synapse function. Furthermore, since the CARMIL3 gene trap did not alter NMDAR-mEPSCs, the AMPAR-mediated reduced frequency must be due to postsynaptic effects, with fewer synapses containing AMPA-type receptors at their surface.

### CARMIL3 interacts with both capping protein and Wrp

**Fig. 7:**
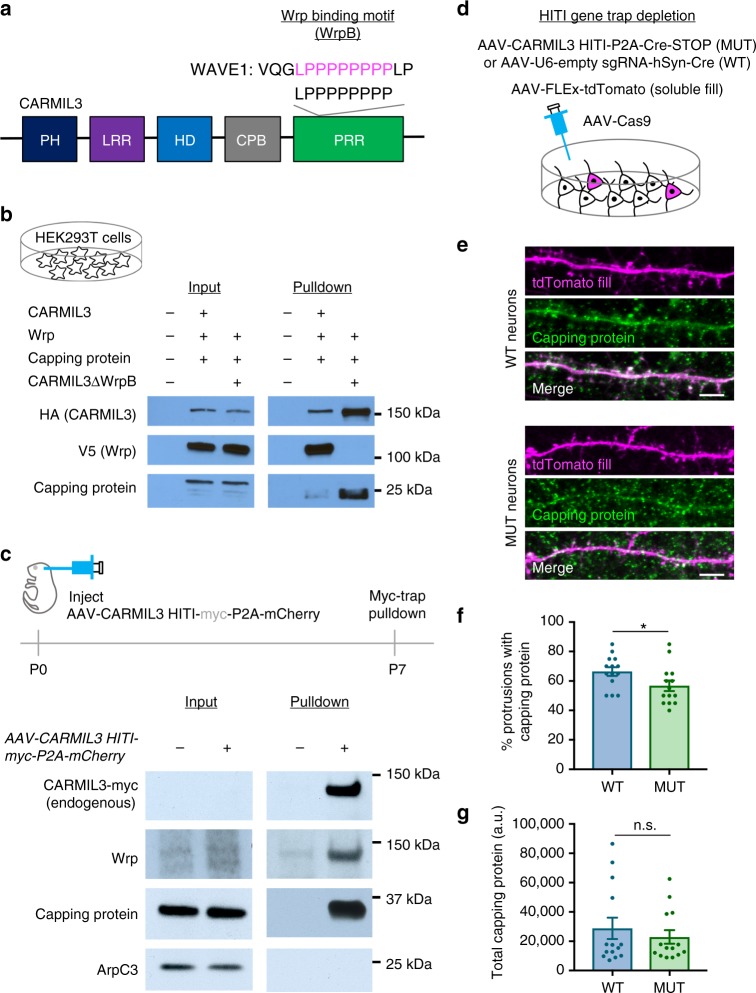
CARMIL3 interacts with both Wrp and capping protein in developing synapses. **a** Schematic depicting domains of CARMIL3. PH pleckstrin homology domain, LRR leucine-rich region, HD helical domain, CPB capping protein-binding domain, PRR proline-rich region. The beginning of the PRR also contains a region with homology to the Wrp-binding motif (WrpB) of WAVE1. **b** Pulldowns of HA epitope-tagged CARMIL3 or CARMIL3 with a deletion of the 8 amino acid Wrp-binding motif. CARMIL3, Wrp, and capping protein constructs were overexpressed in HEK293T cells. **c** Pulldowns of endogenous CARMIL3 with HITI epitope-tagged Myc on the c-terminus. Both Wrp and capping protein, but not ArpC3 co-IP with CARMIL3 in vivo in the mouse forebrain. **d** Schematic depicting CRISPR depletion of CARMIL3 in cultured hippocampal neurons. **e** Representative images of WT and MUT neurons at DIV16 stained for capping protein (green) and tdTomato fill (magenta). Scale bars are 5 μm. **f** Graphical representation of the percentage of dendritic protrusions with capping protein for WT (66 ± 3%, *n*=14 neurons) and MUT (57 ± 4%, *n*=14 neurons) neurons at DIV16. *p*=0.0473. **g** Graphical representation of the total amount of capping protein in WT (28823.00 ± 7271.45 a.u., *n*=14 neurons) and MUT (22943.41 ± 4640.35 a.u., *n*=14 neurons) dendrites at DIV16. Error bars are standard error of the mean (SEM). *p*=0.5026. *p<0.05, *t*-tests

The combined results of our imaging and electrophysiology studies reveal that CARMIL3 is important for aspects of both morphological and functional synaptogenesis, but how CARMIL3 functions at a molecular level was unclear. CARMIL3 contains membrane and protein interaction domains that have been previously characterized in other CARMIL family members CARMIL1–2 (Fig. 7a). Prior functional studies of CARMIL1 demonstrate one of its main cellular functions is to recruit capping protein (CP) to sites of Arp2/3-dependent actin remodeling through a distinctive capping protein-binding domain that is conserved in CARMIL3 (refs. ^22,44^). siRNA knockdown of CP subunit Β2 in cultured neurons leads to a phenotype similar to CARMIL3 depletion, including reduced spine density and mEPSCs^45^, suggesting the possibility that CARMIL3 facilitates CP recruitment and/or activity. We also noted that the CARMIL3 proline-rich region contained a sequence that is homologous to the WRP-SH3 domain-binding motif of WAVE1 (Fig. 7a)^36^, suggesting that in addition to binding CP, CARMIL3 may also directly interact with Wrp.

To first test these possibilities, we expressed V5-epitope tagged Wrp, CP subunit Β2, and HA epitope tagged CARMIL3 or CARMIL3 lacking the predicted Wrp-binding site (CARMIL3 ΔWrp) in HEK293T cells. CARMIL3 immunoprecipitation followed by western blot analysis revealed both Wrp and a small amount of CP co-immunoprecipitated (co-IP) with CARMIL3 when all three were co-expressed. Further, deleting the predicted Wrp-binding motif abrogated the co-IP between Wrp and CARMIL3, demonstrating the interaction between the two requires this motif (Fig. 7b). Interestingly, CARMIL3 ΔWrp was better able to co-precipitate CP, suggesting that there may be competition between Wrp and CP to bind CARMIL3, which is consistent with the juxtaposed binding sites for CP and Wrp in CARMIL3. As predicted from CARMIL1, deletion of the homologous CP-binding region of CARMIL3 abolished CP but not Wrp co-IP (Supplementary Figure 9). Moreover, co-IP between Wrp and CARMIL3 required the Wrp-SH3 domain, as deletion of this domain in WRP showed it was required for the interaction with CARMIL3 (Supplementary Figure 9).

We next determined whether the interactions between CARMIL3, Wrp, and CP occur in vivo between the endogenous proteins. To test this possibility, we used HITI to epitope tag endogenous CARMIL3 with a c-terminal myc epitope by intracortically infusing P0 pups with AAV-CARMIL3 HITI-myc-P2A-mCherry vs negative control uninfected pups (Fig. 7c, top). At P7, whole brain lysates were prepared (input) and immunoprecipitated with myc nano-trap resin. While the expression level of endogenous CARMIL3-myc was too low to be detected in lysates, immunopurification resulted in a strong myc-immunoreactive band at the predicted 150 kDa size for CARMIL3 in HITI-myc-infected brain tissue. Western blot analysis of CARMIL3-myc vs negative control IP revealed that CARMIL3-myc co-precipitated with both endogenous Wrp and CP (Fig. 7c, middle panels). The ArpC3 subunit of the Arp2/3 complex did not co-precipitate with CARMIL3-myc, consistent with prior work suggesting the mammalian CARMIL proteins do not interact with Arp2/3 (ref. ^21^) (Fig. 7c, bottom panel).

Previous work has shown that for CARMIL1, the recruitment of CP to sites of actin remodeling is one of its important cellular functions^21,22,44^. Further, CP facilitates the generation of Arp2/3-dependent branched actin filament networks^23^ and both Arp2/3 and CP are critical for spinogenesis^5,45^. As our data demonstrate CARMIL3 interacts with CP, we hypothesized that CARMIL3 may be required for facilitating the localization of CP to dendritic spines. To test this hypothesis, we depleted CARMIL3 using the CARMIL3 HITI gene trap strategy and immunostained for endogenous CP (Fig. 7d, e). Quantification of the percentage of dendritic spine protrusions immunoreactive for CP revealed a slight but significant decrease in CARMIL3 gene-trapped neurons when compared to control wild-type neurons (Fig. 7f). The total levels of capping protein in dendrites were unaltered (Fig. 7g). Thus, CARMIL3 binds CP and appears to facilitate CP localization to dendritic protrusions, which is known to be important for spinogenesis.

## Discussion

Here we report the proximity-based in vivo biotinylation (iBioID) of dendritic spine proteomes during their initial formation. The resulting quantitative proteomic analysis discovered 60 early synaptic candidate proteins. Remarkably, none of the proteins we report here were previously identified in prior proteomic studies of spine development based on traditional fractionations that are probably optimal for fully mature synapses rather than early developing synapses^46,47^. Consistent with this, over half of the proteins we identified were not identified in prior proteomic studies of mature synapses, yet these unique proteins are predicted to mediate synapse adhesion and synapse formation. Thus, iBioID, by covalently labeling nascent synaptic proteins based on their proximity to probes targeted to these structures, is able to access proteomes difficult to enrich by traditional biochemical approaches. We validated our proteomics screen by utilizing CRISPR-Cas9 to target and deplete four candidates and found that three out of four altered the morphological formation of dendritic protrusions during spinogenesis. Labeling of endogenous proteins by HITI also strongly supported that our proteomics analysis identified regulators of dendritic spine formation. Thus, iBioID can be used for proximity biotinylation of key neurodevelopmental milestones in vivo.

Dendritic spine maturation is one key milestone whose dysregulation is heavily implicated in neurodevelopmental disorders^10,14–17,48,49^. We note that 21% of the proteins we discovered in developing synapses either may be regulated by FMRP (Fragile X Mental Retardation Protein)^50^ (*Ptpn11*, *Lrrc7*, *Lphn1*, *Lphn3*, *Grin1*, *Grin2b*, *Dab2ip*, *Ntrk2*, *Odz4*, *Grm5*, *Pkp4*) or are directly implicated by gene mutations in neurodevelopmental disorders, including *Erbb2ip*^51,52^, *Cnksr2* (refs. ^53,54^), and *Wrp* (also known as *Srgap3)*^55^. Interestingly, both Cnksr2 and Wrp are thought to modulate Rac-dependent signaling events that instruct actin cytoskeletal remodeling^56–58^, which was also well-represented in the Wrp-BirA proteome dataset (Fig. 2e). One of the novel cytoskeletal proteins we discovered not previously implicated in spinogenesis was *Lrrc16b*, which encodes CARMIL3. In our initial CRISPR-based screen we found targeting of *Lrrc16b*/CARMIL3 significantly reduced dendritic protrusions during the period of synaptogenesis, suggesting it is important for modulating excitatory synapse formation.

Little is known about CARMIL3, though its close family member CARMIL1 binds CP and regulates cellular actin filament networks^21,22^. Thus, a key question was, how might CARMIL3 regulate the maturation of dendritic spines in developing neurons mechanistically? First, using HITI to tag endogenous CARMIL3 with antibody epitopes and track its expression via co-translation of mCherry, we found that CARMIL3 is expressed in cortical and hippocampal neurons during synaptogenesis and that it is localized to discrete patches along the dendritic shaft and filopodia or early spines. Thus, CARMIL3 protein is spatially and temporally positioned to modulate synapse formation.

The notion that CARMIL3 localizes to synapses is further supported by our finding that it physically interacts with Wrp. We identified a motif in the proline-rich c-terminal region of CARMIL3 that is highly similar to the Wrp-SH3 domain binding motif of WAVE1^56^. Our co-IP data and deletion mutagenesis analysis revealed that CARMIL3 associates with Wrp through this Wrp-binding motif and the SH3 domain of Wrp. We speculate that the interaction between CARMIL3 and Wrp further localizes CARMIL3 not just to the membrane, but to developing synapses as Wrp is highly enriched in dendritic filopodia and dendritic spines^11,18^.

Finally, we found endogenous CARMIL3 co-IPs with neuronal capping protein (CP) and our deletion mutagenesis data suggest that this interaction occurs via a CP-binding motif conserved in CARMIL1^20,21,41,59,60^. CP strongly influences actin cytoskeletal remodeling by binding to the barbed ends of actin filaments and thus limits their linear growth by preventing the addition of monomeric actin. Using in vitro assays with purified proteins, conflicting reports suggest CARMIL1 can either allosterically inhibit CP or weakly activate CP^20,21,44,60–62^.

In cellular assays the role of the interaction between CARMIL family members and CP appears clearer. With CARMIL1 localizing predominately to membranes undergoing active protrusions^44^, CARMIL1 also promotes actin network assembly by enhancing barbed-end capping activity^63^. CARMIL family proteins are unlike other allosteric CP inhibitors in that CARMIL family members interact with CP at a region that is distinct from its actin-binding site^21^, strongly suggesting that they function to localize CP to sites of cellular actin dynamics. This finding is consistent with models proposing that capping of actin filaments leads to the biased binding of actin to Arp2/3, transforming actin filaments from linear to highly branched actin networks^23,25^. During spinogenesis, filopodia transition to spines composed of highly branched networks of actin. Our data suggest that CARMIL3, via its ability to recruit capping protein during synaptogenesis, facilitates Arp2/3 activity, which we have previously demonstrated is also required for spine morphogenesis^5^.

**Fig. 8:**
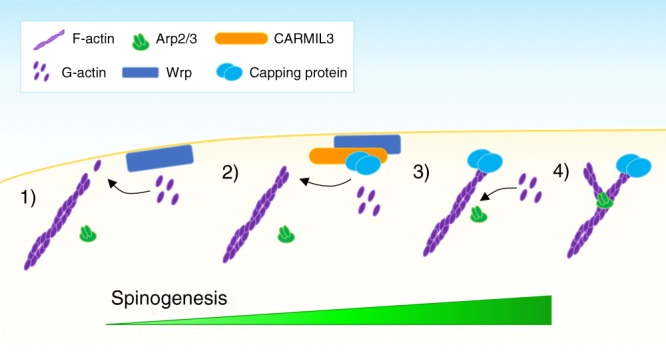
Schematic of proposed CARMIL3 function during synapse development. (1) In the absence of CARMIL3 and capping protein, monomeric (G-actin) is preferentially added to the barbed (+) ends of filamentous actin (F-actin). (2) CARMIL3 bound to capping protein is recruited to the membrane of developing protrusions through its interaction with Wrp. (3) Capping protein binds to the barbed ends of actin filaments, preventing the addition of G-actin. (4) G-actin is now primed for nucleation by the Arp2/3 complex, which shifts the actin dynamics of the developing synapse from largely linear actin to a dense, branched actin network required for synaptic maturation

The likelihood that CARMIL3-dependent recruitment of CP facilitates Arp2/3 activity during spinogenesis is further supported by our finding that depletion of CARMIL3 results in reduced AMPAR- but not NMDAR-mediated postsynaptic currents. We recently found that loss of Arp2/3-dependent actin remodeling also impairs the recruitment of AMPAR, but not NMDAR, during spine development^5^. Furthermore, depletion of CP also results in fewer spines and a reduced frequency of mEPSCs^45^. Together, the current and prior studies suggest a new mechanistic model in which spine development and AMPAR unsilencing is facilitated by a CARMIL3 and CP complex that may potentiate Arp2/3 activity, transitioning filopodia to fully functional spine synapses (Fig. 8).

In summary, by leveraging iBioID to discover local proteomes enriched within developing excitatory postsynapses, we have uncovered a rich diversity of receptors, signaling, and adhesion molecules uniquely positioned to promote synaptogenesis. Our analysis of one novel protein we found, CARMIL3, demonstrates that it plays a specific role in spinogenesis by localizing CP to developing synapses, which we speculate triggers Arp2/3-dependent transitions to both morphologically and functionally mature spines. Future studies of this proteome during early development hold promise for revealing new insights in normal and maladaptive mechanisms of excitatory synaptic transmission.

## Methods

### Animals

*Lox-stop-Lox*-Cas9-P2A-GFP (Cas9 KI) mice (Jax# 026175) and C57BL/6J (WT) mice (Jax# 000664) were purchased from Jackson laboratory. CMV-Cre mice were previously published^64^ and Cre was subsequently outbred prior to experimental analysis. All mice were housed (2–5 mice per cage) in Duke University′s Division of Laboratory Animal Resources facilities. All procedures were conducted with a protocol approved by the Duke University Institutional Animal Care and Use Committee in accordance with US National Institutes of Health guidelines.

### Constructs

For a complete list of constructs, see Supplementary Table 1. pSpCas9(BB)-P2A-GFP (PX458) (Addgene plasmid # 48138)^65^, PX551(Addgene plasmid # 60957)^66^, and pX330-U6-Chimeric_BB-CBh-hSpCas9 (Addgene plasmid # 42230)^65,66^ were a gift from Feng Zhang. CAPZB2-TwinStrepII was a gift from Peter Barr-Gillespie (Addgene plasmid # 83194). pAAV-FLEx-tdTomato was a gift from Edward Boyden (Addgene plasmid # 28306). All constructs generated in the Soderling lab were validated with sequencing (Eton).

### AAV viruses

For a complete list of viruses, see Supplementary Table 2. AAV-hSynI-BirA-HA, AAV-hSynI-MT-BirA-HA, AAV-hSynI-Wrp-BirA-HA, and AAV-CAG-FLEx-tdTomato were generated by the University of Pennsylvania Vector Core. All other viruses were produced in the Soderling lab using iodixanol (OptiPrep; Sigma) gradients^18^.

### Cell lines

HEK293T cells were obtained from the Duke Cell Culture Facility and used to prepare AAV viruses and for recombinant protein expression in co-immunoprecipitation. HEK293T cells were chosen as they are commonly used for both of these applications.

### Primary neuronal culture

Hippocampi from P0 pups were dissected and incubated with papain (Worthington) at 37 °C for 18 min Then, neurons were dissociated by trituration and plated onto poly-l-lysine (Sigma)-treated coverslips. Neurons were maintained in Neurobasal A medium supplemented with 2% (v/v) B-27 supplement and 1% (v/v) GlutaMAX (Invitrogen) in an incubator at 37 °C and 5% CO_2_. On DIV5, 5 μM cytosine arabinoside (Sigma) was added to inhibit glial division. Neuronal transfections were performed with Lipofectamine 2000 (Invitrogen) according to the manufacturer′s instructions on DIV5 after plating, with the modification that neurons were only incubated with lipofectamine for 30 min. Neuronal electroporations were performed before plating cultured neurons at DIV0 (Amaxa) and were performed according to the manufacturer’s instructions.

### Immunocytochemistry

Neurons were fixed at indicated time points in 4% PFA/4% sucrose for 10 min at 37 °C. They were permeabilized with 0.2% Triton X-100 and blocked with blocking buffer (Abcam, ab126587) for 1 h at room temperature. Samples were then incubated for 1 h at room temperature with primary antibodies: rabbit anti-RFP (Rockland, 600-401-379, 1:1000), Homer1 (Synaptic Systems, 160002,1:2000), mouse anti-HA (Biolegend, 16B12, 1:1000), rat anti-HA (Sigma, 3F10, 1:200), chicken anti-GFP (Abcam, ab13970, 1:1000), mouse anti-PSD-95 (ThermoFisher, MA1-046, 1:500), rabbit anti-capping protein beta (Millipore, AB6017, 1:500), and rat anti-red (Chromotek, 5F8, 1:1000). After washing three times with phosphate-buffered saline (PBS), samples were incubated for 1 h at room temperature in secondary antibodies: Alexa Fluor 555 Goat anti-Rabbit (ThermoFisher, A21428, 1:1000), Alexa Fluor 488 Streptavidin (ThermoFisher, S32354, 1:500), Alexa Fluor 647 Goat anti-Mouse (ThermoFisher, A32728, 1:1000), Alexa Fluor 488 Goat anti-Chicken (ThermoFisher, A-11006, 1:1000), Alexa Fluor 647 Goat anti-Rat (ThermoFisher, A-21247, 1:500), Alexa Fluor 488 Goat anti-Rabbit (ThermoFisher, A-11034, 1:1000), Alexa Fluor 568 Goat anti-Rat (ThermoFisher, A-11077, 1:1000). Samples were washed three times with PBS and mounted with mounting media (Fluorsave Reagent, EMD Millipore, 345789). Mounted coverslips were imaged on a Zeiss LSM 710 confocal microscope.

### iBioID probe intensity analysis

Hippocampal neurons were created, cultured, and immunostained as described above. All images were acquired by z-series (0.13 µm intervals) using a ×63/1.4 numerical aperture (NA) oil-immersion objective. Images were then analyzed in ImageJ. Intensity for both streptavidin as well as HA signal was measured in both dendrites as well as dendritic protrusions. Enrichment was calculated by dividing the intensity for either HA or streptavidin in dendritic protrusions by the respective fluorescent intensity of the corresponding dendrite. Dendritic protrusions were defined as previously published^11^, with filopodia defined as protrusions where the head/tip was less than twice the width of the spine neck. Protrusions were defined as spines when the head tip was more than or equal to twice the width of the spine neck.

### Developmental in vivo BioID (iBioID)

Pregnant WT female mice were injected with 24 mg/kg biotin starting at E17.5, approximately 3 days before delivering a litter of pups. When pups were delivered, they were intracranially infused with the AAV BirA probe viruses predominately into the hippocampus and cortex at P0. Pups and dams also received daily 24 mg/kg biotin injections from P0 to P5. At P5, the brain (excluding the cerebellum) was collected. For each probe, five mice were used for each round of purifications. A total of three purification rounds were performed independently. iBioID purification^18^ was performed using capture of biotinylated proteins with high capacity NeutrAvidin affinity resin (Pierce), extensive serial washing with: 2% sodium dodecyl sulfate (SDS); 1% Triton X-100, 1% deoxycholate, 25 mM LiCl; 1 M NaCl; and 50 mM Ambic. Samples were eluted at 60 °C in 0.1% RapiGest/2 mM biotin/50 mM Ambic.

### Differential protein expression analysis by LC-MS/MS

The Duke Proteomics Core Facility received nine eluents from streptavidin resins. All samples were lyophilized to dryness and resuspended in 20 μL of LDS loading buffer supplemented with 10 mM DTT. All 20 μL was loaded onto an Invitrogen NuPAGE 4–12% SDS-polyacrylamide gel electrophoresis (PAGE) gel and run for approximately 5 min to electrophorese all proteins into the gel matrix. The entire MW range was then excised in a single gel-band and subjected to standardized in-gel reduction, alkylation, and tryptic digestion. Following lyophilization of the extracted peptide mixtures, samples were resuspended in 12 μL of 2% acetonitrile/1% TFA supplemented with 10 fmol/μL yeast ADH. From each sample, 3 μL was removed to create a QC Pool sample which was run periodically throughout the acquisition period.

Quantitative LC/MS/MS was performed on 3 μL of each sample, using a nanoAcquity UPLC system (Waters Corp) coupled to a Thermo QExactive Plus high-resolution accurate mass tandem mass spectrometer (Thermo) via a nanoelectrospray ionization source. Briefly, the sample was first trapped on a Symmetry C18 300 mm×180 mm trapping column (5 L/min at 99.9/0.1 v/v water/acetonitrile), after which the analytical separation was performed using a 1.7 um Acquity BEH130 C18 75 mm × 250 mm column (Waters Corp.) with a 90-min linear gradient of 5–40% acetonitrile with 0.1% formic acid at a flow rate of 400 nL/min with a column temperature of 55 °C. Data collection on the QExactive Plus mass spectrometer was performed in a data-dependent acquisition (DDA) mode of acquisition with a *r*=70,000 (@ *m*/*z* 200) fμLl MS scan from *m*/*z* 375 to 1600 with a target AGC value of 1e6 ions followed by 10 MS/MS scans at *r*=17,500 (@ *m*/*z* 200) at a target AGC value of 5e4 ions. A 20 s dynamic exclusion was employed to increase depth of coverage. The total analysis cycle time for each sample injection was approximately 2 h. Sample order of data collection was interwoven between conditions in order to minimize temporal bias.

Following the 12 LC-MS/MS analyses, data were imported into Rosetta Elucidator v3.3 (Rosetta Biosoftware, Inc.), and all LC-MS/MS runs were aligned based on the accurate mass and retention time of detected ions (“features”) which contained MS/MS spectra using PeakTeller algorithm (Elucidator) and intensity-scaled based on a robust mean (10%) of the feature intensity within a sample. The relative peptide abundance was calculated based on area under-the-curve of aligned features across all runs. The overall dataset had 73,343 quantified isotope (peptide) groups. Additionally, 380,251 MS/MS spectra were acquired for peptide sequencing by database searching. These MS/MS data were searched against a SwissProt_Mouse database which also contained a reversed-sequence “decoy” database for false-positive rate determination. Database searching was performed within Mascot Server (Matrix Science) and then subjected to peptide/protein teller algorithm within Rosetta Elucidator to annotate the data at a ~0.9% peptide level FDR. Searching allowed variable M (oxidation, +16 Da) and NQ (deamidation, +1 Da) modifications. Carbamidomethylation on C (alkylation, +57 Da) was considered static modifications.

Due to the inherent variability in immunoprecipitation efficiency, carboxylases and keratins were removed and then the remaining signals were scaled to a robust mean (excluding top and bottom 10% of the signals). To assess system variability, the intensity scaled for all non-carboxylase/keratin proteins (*n*=2463) across three QC Pool samples was calculated to give instrument (LC-MS/MS) system reproducibility. This QC metric resulted in an average CV (relative standard deviation) of 22.7% and a median %CV of 15.4%. To assess preparation variation across the same *n*=2463 proteins, the intensity-scaled protein expression data from all non-carboxylase/keratin proteins within WRP, MT, and BirA groups were calculated to be 35.8%, 43.4%, and 29.5%, respectively. To identify proteins specific to the WRP group vs the other two controls, two sets of two-tailed *t*-test were performed on log2 protein intensities: WRP vs MT and WRP vs BirA. Fold changes were calculated for the same comparisons by ratioing the average protein intensity in one group vs the other. To consider something specific for WRP interaction or MT interaction, we required a *p*-value <0.05 and fold changes greater than 2.5 in WRP samples or MT samples vs the negative control, BirA alone.

### CRISPR guide validation assay

To select guides to use for CRISPR screening, the first or second coding exon was submitted to the CRISPR Design Tool website (crispr.mit.edu). Three guide sequences were selected from top scoring candidates and cloned into vector PX458 to create the depletion vectors for a gene of interest. The genomic target sequence was also cloned directly in front of and in frame with the GFP in the pBA-GFP vector to create a GFP validation vector for each guide. HEK293T cells were then PEI transfected with PX330 (Cas9), a depletion vector (or unmodified PX458 without a guide sequence as a control), and the corresponding GFP validation vector. After 48 h cells were either collected for depletion validation by western blot (LRRC7, LPPR4, and LRRC16B) or fluorescent imaging (LAP2). To validate depletion by western blot, HEK cells were lysed in RIPA buffer and rocked on ice for 30 min. Cells were then spun down for 30 min at 12,000 × *g* at 4 °C in a tablevtop centrifuge. Supernatant protein density was then calculated by bicinchoninic acid assay (BCA). Equal protein densities were run out on an SDS-PAGE gel (Bio-rad, 4561083) and then transferred onto a nitrocellulose membrane. Successful transfer was verified with a Ponceau stain and membrane was blocked in fluorescent blot blocking buffer for 1 h (Rockland, MB-070). Membrane was then incubated overnight in Mouse anti-Β-actin (Novus, 501ss, 1:5000) and Rabbit anti-GFP (ThermoFisher, a11122, 1:1000). Membrane was washed three times for 10 min in TBST and then incubated for 2 h in Alexa Fluor 680 goat anti-mouse (ThermoFisher, A32729, 1:5000) and Alexa Fluor 680 goat anti-Rabbit (ThermoFisher, A32734, 1:5000). Proteins were detected using Li-Cor Odyssey Infrared Imaging System and quantified in ImageJ. Original scans of blots are included (Supplementary Figure 10). To validate depletion by fluorescence imaging, cells were fixed in 4% PFA for 10 min at 37 °C and stained with DAPI. Cells imaged on a ×5 epifluorescence microscope (Zeiss) and DAPI staining was used to validate that cell densities remained consistent for each field of view. GFP intensity was analyzed in ImageJ by calculating the corrected total cellular fluorescence (CTCF). CTCF=integrated fluorescence density−(area of cells × mean background fluorescence). Of the guides tested for each construct, the guide with the lowest GFP expression was selected for depletion assays.

### CRISPR/Cas9 screening of proteome candidates

AAV depletion viruses were made for each candidate (CARMIL3, LPPR4, LRRC7, and LAP2) utilizing the most efficient guide from the GFP-depletion assay described above. Hippocampal neurons cultures were created from P0 Cas9 KI mouse pups as described above and cultures were infected with either a control virus (PX458, no guide) or a depletion virus, as well as AAV-FLEx-tdTomato. Neurons were fixed, immunostained, and imaged between DIV14 and 16 as described above. Images were then analyzed in ImageJ with dendritic protrusions defined as filopodia when the head/tip was less than twice the width of the spine neck and as spines when the head/tip was equal to or more than twice the width of the spine neck. These experiments were conducted blinded to the condition.

### HITI-mediated epitope tagging of endogenous proteins

In order to epitope tag the c-terminus of proteins using HITI^26^, guides were chosen close the end of the coding region. Following the cut site, the remaining amino acids of CARMIL3 as well as the epitope tag (either myc or smFP) were inserted, followed by a P2A sequence and mCherry to create a soluble fluorescent mark of cells positive for CARMIL3 tagging or smFP was directly inserted in-frame (Lrrc7 and Lppr4).

### HITI-mediated pulldown of endogenous CARMIL3-myc

AAV-U6-CARMIL3Ctermsg1-HITI-myc-P2A-mCherry-SynI-Cre virus was intracortically infused into P0 Cas9 KI mice. Brain samples were collected at P7, P14, P21, and P28. Samples were homogenized in RIPA buffer, transferred to an epi tube, and spun down at max speed for 30 min on a tabletop centrifuge at 4 °C. Supernatant was removed and was precleared with protein G agarose beads for 1 h. Beads were removed, and supernatant protein density was quantified with BCA. Equal protein volumes were added to anti-Myc beads (Chromotek, yta-10) and incubated overnight. Supernatant was removed, and beads were washed with 1 mL of IP Wash Buffer (10 mM Tris-CL, 150 mM NaCl, 1% Triton X-100, 1 mM EDTA) for 10 min two times. Proteins were eluted off beads with 2× Sample Buffer incubated at 95 °C for 5 min. Equal protein densities were run out on an SDS-PAGE gel (Bio-rad, 4561083) and then transferred onto a nitrocellulose membrane. Successful transfer was verified with a Ponceau stain and membrane was blocked in fluorescent blot blocking buffer for 1 h (Rockland, MB-070) or Blocking Buffer (1% bovine serum albumin (BSA), 2% milk). Membrane was then incubated overnight in Rat anti-HA (Sigma, 3F10, 1:2000). Membrane was then washed three times with TBST for 10 min each wash and then incubated with either Alexa Fluor Goat anti-Rat (ThermoFisher, A-21096, 1:5000) or HRP anti-Rat (GE Healthcare Life Sciences, NA935, 1:5000). Blots were either imaged using the Li-Cor Odyssey Infrared Imaging System or autoradiography film (Genesee Scientific, 30–100). Original scans of blots are included (Supplementary Figure 10).

### HITI-mediated labeling of CARMIL3-smFP in whole brain

AAV-U6-CARMIL3 Cterm sg1-HITI-smFP-P2A-mCherry-SynI-Cre virus was intracortically infused in P0 Cas9 KI mice as described above. Mice were perfused at P14 and brains were cryosectioned to 40 μm sections. Brain slices were then permeabilized for 2 h in 0.2% Triton X-100 and then blocked for 2 h in blocking buffer (Abcam, ab1265870). Slices were then incubated in primary antibodies: Rat Anti-HA (Sigma, 3F10, 1:200), Chicken Anti-GFP (Abcam, ab13970, 1:1000), and Rabbit anti-RFP (Rockland, 600-041-379, 1:1000) for 72 h. Slices were then washed three times in PBS for hour each wash and incubated in secondary antibodies: Alexa Fluor 647 Goat anti-Rat (ThermoFisher, A-21247, 1:1000), Alexa Fluor 488 Goat anti-Chicken (ThermoFisher, A-11006, 1:1000), and Alexa Fluor 555 Goat anti-Rabbit (ThermoFisher, A32732, 1:1000) for 24 h. Slices were then washed three times in PBS for hour each wash and mounted on slides. Slices were imaged and tiled on a Zeiss LSM 710 confocal microscope.

### HITI-mediated labeling of CARMIL3-smFP for localization

Cultured hippocampal neurons were created from WT P0 pups as described above. Neurons were infected at DIV0–DIV1 with AAV-U6-CARMIL3Ctermsg1-HITI-smFP-P2A-mCherry-SynI-Cre and also either infected with pAAV-pMecp2-SpCas9-spA (AAV-Cas9), PX551, or lipofectamine transfected at DIV2 with PX330 (Cas9). At DIV14–16, neurons were fixed in and prepped for primary antibody incubation as described above. Neurons were incubated in primary antibodies: Rat Anti-HA (Sigma, 3F10, 1:200), Chicken Anti-GFP (Abcam, ab13970, 1:1000), and Rabbit anti-RFP (Rockland, 600-041-379, 1:1000) for 16–18 h at 4 °C. Neurons were then washed three times in PBS for 5min each and incubated in secondary antibodies: Alexa Fluor 647 Goat anti-Rat (ThermoFisher, A-21247, 1:1000), Alexa Fluor 488 Goat anti-Chicken (ThermoFisher, A-11006, 1:1000), and Alexa Fluor 555 Goat anti-Rabbit (ThermoFisher, A32732, 1:1000) for 1–2 h. Coverslips were then mounted with mounting media (Fluorsave Reagent, EMD Millipore, 345789) and imaged on a Zeiss 880 Airyscan inverted confocal microscope.

### CARMIL3 morphology analysis

Cultured neurons were created from Cas9 KI P0 pups as described above. Neurons were infected with AAV-CAG-FLEx-tdTomato and either AAV-U6-(empty sgRNA)-hSynI-Cre (Control) or AAV-U6-CARMIL3sg1-hSynI-Cre (MUT) virus at DIV0–DIV1 for depletion analysis or DIV10 for late depletion analysis. Neurons were fixed and prepped for immunostaining as described above at either DIV8 or DIV14–16. Neurons were immunostained with Chicken Anti-GFP (Abcam, ab13970, 1:1000) and Rabbit anti-RFP (Rockland, 600-041-379, 1:1000) for 1 h at room temperature. Neurons were then washed three times in PBS for 5min each and incubated in secondary antibodies: Alexa Fluor 488 Goat anti-Chicken (ThermoFisher, A-11006, 1:1000) and Alexa Fluor 555 Goat anti-Rabbit (ThermoFisher, A32732, 1:1000) for 1 h. Coverslips were then mounted with mounting media (Fluorsave Reagent, EMD Millipore, 345789) and imaged on a Zeiss 710 inverted confocal microscope. Images were analyzed in ImageJ. Dendritic protrusions were defined as previously published^5,11^, with filopodia defined as protrusions where the head/tip was less than twice the width of the spine neck. Protrusions were defined as spines when the head tip was more than or equal to twice the width of the spine neck. These experiments were conducted blinded to the condition.

### CARMIL3 gene trap morphology analysis

HITI constructs were designed as described previously^26^. In order to delete CARMIL3 and conditionally label neurons only where CARMIL3 had been depleted, the depletion guide from morphology analysis was used. Following the cut site, which interrupts CARMIL3 in the first coding exon, a P2A sequence followed by Cre and a STOP codon was inserted. This allows for the expression of Cre under the CARMIL3 promoter only when CARMIL3 has been replaced with Cre. P0 Cas9 KI mice that had been crossed with a CMV-Cre mouse (in order to express Cas9 constitutively in all cells) were intracortically infused with the CARMIL3 gene trap virus (AAV-U6-CARMIL3sg1-HITI-P2A-Cre-STOP) as well as AAV-CAG-FLEx-tdTomato. Control WT mice were injected with AAV-Cre-ERT2 and AAV-CAG-FLEx-tdTomato and then injected with tamoxifen at P7 to get sparse labeling as our lab had done previously^10^. At P14, mice were perfused, the brains were cryosectioned, and prepped for immunostaining as described above. Slices were incubated in primary antibody: Rabbit anti-RFP (Rockland, 600-041-379, 1:1000) for 72 h. Slices were then washed three times in PBS for hour each wash and incubated in secondary antibody: Alexa Fluor 555 Goat anti-Rabbit (ThermoFisher, A32732, 1:1000) for 24 h. Slices were then washed three times in PBS for hour each wash and mounted on slides. Slices were imaged and tiled on a Zeiss LSM 710 confocal microscope. Morphology was analyzed in ImageJ as described above.

### Electrophysiology

Somatic whole-cell currents were recorded from cultured hippocampal neurons on DIV12–16 under a Zeiss Axio Examiner.D1 microscope. Patch pipettes (4–7 MΩ) were created from borosilicate glass capillaries (Sutter Instrument) using a P-97 puller (Sutter Instrument). Pipette intracellular solution contained 135 mM Cs-methanesulfonate, 10 mM HEPES, 10 mM phosphocreatine, 8 mM NaCl, 5 mM TEA-Cl, 5 mM QX-314, 4 mM MgATP, 0.3 mM EGTA, and 0.3 mM Na2GTP (pH 7.3 with CsOH, 295 mOsm/L). Coverslips were superfused with artificial CSF (aCSF) containing 124 mM NaCl, 26 mM NaHCO_3_, 10 mM dextrose, 4 mM CaCl_2_, 3 mM KCl, 1.3 mM MgSO_4_, 1.25 mM NaH_2_PO_4_, and 0.5 μM TTX (310 mOsm/L), continuously bubbled at room temperature with 95% O_2_ and 5% CO_2_. AMPAR-mEPSCs were recorded at −70 mV holding potentials in aCSF with the following modifications: 100 μM picrotoxin, 10 μM bicuculline methiodide, and 50 μM D-AP5. NMDAR-mEPSCs were recorded at −55 mV holding potentials in aCSF with the following modifications: 0 mM MgSO_4_, 100 μM picrotoxin, 10 μM bicuculline methiodide, 20 μM CNQX, 50 μM glycine, and 2 μM strychnine. mIPSCs were recorded at 0 mV holding potentials in aCSF with the following modifications: 2 mM CaCl_2_, 50 μM D-AP5, and 20 μM CNQX. No corrections were made for the 8.5–9.0 mV estimated liquid junction potentials of these solutions. Series resistance was monitored throughout all recordings with brief 5 mV hyperpolarizing pulses, and only recordings which remained stable over the period of data collection were used. Data were recorded with a Multiclamp 700B amplifier (Molecular Devices), digitized at 50 kHz with a Digidata 1550 (Molecular Devices), and low-pass filtered at 1 kHz. Five hundred currents per cell were manually detected offline using MiniAnalysis (Synaptosoft) with a detection threshold of 5 pA. Decay time constants were determined from mono-exponential fits of the average current decay using Clampfit (Molecular Devices). All drugs were purchased from Sigma-Aldrich or Tocris. These experiments were performed blinded to the condition.

### Superecliptic pHlourin (SEP) imaging of surface GluA1 and GluA2

Before plating, cultured neurons were electroporated with SEP-GluA1 and SEP-GluA2. Control and HITI gene trap viruses were added on DIV0 as described above. On DIV12–16, neurons were imaged live on a Zeiss LSM 710 inverted confocal microscope in HEPES-buffered aCSF containing (in mM) 130 NaCl, 20 HEPES, 2 NaHCO_3_, 25 d-glucose, 2.5 KCl, and 1.25 NaH_2_PO_4_, pH 7.35. Fluorescence intensity was analyzed from background-subtracted images in ImageJ. Protrusions were classified as spines or filopodia as described earlier using the tdTomato fluorescence. SEP fluorescence was measured in an ROI at the tips of protrusions and their base in dendritic shafts. At least five protrusions were analyzed per dendrite. The analysis was conducted blinded to the condition.

### HEK293T cell CARMIL3 co-IPs

HEK293T cells were PEI transfected with pBA-HA-CARMIL3 or pBA-HA-CARMIL3ΔWrpBR or pBA-HA-CARMIL3ΔCPB and either EF1alpha-Wrp (full-length) or EF1alpha-Wrp-deltaSH3 and CAPZB2-TwinStrepII. Untransfected HEK cells were used as a negative control. After 48 h cells were either collected for CARMIL3 pulldown. HEK cells were lysed in IP lysis buffer (25 mM HEPES, 150 mM NaCl, 0.5% Triton X-100, and 1 mM EDTA) and rocked on ice for 30 min. Cells were then spun down for 30 min at 12,000 × *g* at 4 °C in a tabletop centrifuge. Supernatant protein density was then calculated by BCA. Equal protein densities were added to Mouse anti-HA beads (Sigma, A2095) and incubated while rotating overnight. Supernatant was removed, and beads were washed with 1 mL of IP Wash Buffer (10 mM Tris-CL, 150 mM NaCl, 1% Triton X-100, 1 mM EDTA) for 10 min two times. Proteins were eluted off beads with 2× Sample Buffer incubated at 95 °C for 5 min. Equal protein densities were run out on an SDS-PAGE gel (Bio-Rad, 4561083) and then transferred onto a nitrocellulose membrane. Successful transfer was verified with a Ponceau stain and membrane was blocked in blocking buffer (1% BSA, 2% milk). Membrane was then incubated overnight in Rat anti-HA (Sigma, 3F10, 1:2000), Mouse anti-V5 (ThermoFisher, R960-25, 1:1000), and rabbit anti-capping protein beta (Millipore, AB6017, 1:500) at 4 °C. Membrane was then washed three times with TBST for 10 min each wash and then incubated HRP anti-Rat (GE Healthcare Life Sciences, NA935, 1:5000), HRP anti-Mouse (GE Healthcare Life Sciences, NA931-VS), and HRP anti-rabbit (GE Healthcare Life Sciences, NA934-100ΜL). Blots were developed using autoradiography film (Genesee Scientific, 30–100). Original scans of blots are included (Supplementary Figure 10).

### HITI-mediated in vivo co-IPs

P0 Cas9 KI pups were intracortically injected with AAV-U6-CARMIL3Ctermsg1-HITI-myc-P2A-mCherry-SynI-Cre as described above. At P7 samples were collected and incubated with Myc beads, run out on an SDS-PAGE gel, transferred, and prepped for immunoblotting as described above. Blots were incubated overnight in Rat anti-HA (Sigma, 3F10, 1:2000), rabbit anti-Wrp (V0111)^56^, and rabbit anti-capping protein beta (Millipore, AB6017, 1:500) at 4 °C. Membrane was then washed three times with TBST for 10 min each wash and then incubated HRP anti-Rat (GE Healthcare Life Sciences, NA935, 1:5000) and HRP anti-rabbit (GE Healthcare Life Sciences, NA934-100ΜL). Blots were developed using autoradiography film (Genesee Scientific, 30–100). Original scans of blots are included (Supplementary Figure 10).

### Capping protein recruitment in CARMIL3-depleted neurons

Control and HITI gene trap viruses were added on DIV0 as described above. Neurons were fixed and prepped for immunostaining as described above at DIV14–16. Neurons were immunostained with rabbit anti-capping protein beta (Millipore, AB6017, 1:500), and Rabbit anti-RFP (Rockland, 600-041-379, 1:1000) overnight at 4 °C. Neurons were then washed three times in PBS for 5min each and incubated in secondary antibodies: Alexa Fluor 488 Goat anti-Rabbit (ThermoFisher, A-11034, 1:1000) and Alexa Fluor 555 Goat anti-Rabbit (ThermoFisher, A32732, 1:1000) for 1 h. Coverslips were then mounted with mounting media (Fluorsave Reagent, EMD Millipore, 345789) and imaged on a Zeiss LSM 710 inverted confocal microscope. Images were analyzed in ImageJ for the presence or absence of capping protein in dendritic protrusions. Total levels of capping protein in dendrites were analyzed using z-projections of the sum of fluorescence. An ROI containing the dendrites was creating by thresholding the tdTomato fluorescence and capping protein fluorescence was measured inside this dendrite region. The corrected total fluorescence was calculated as CTCF=integrated fluorescence density−(area of dendrites × mean background fluorescence). Two dendrites were measured per neuron. The analysis was conducted blinded to the condition.

### Replication and statistics

All *n* values are from at least three independently replicated experiments or biological samples such as mice or cultures. Sample sizes were generally a default of 3; however, if a larger sample size was utilized, a power analysis was performed following three replicates. Descriptive and inferential statistics were performed in Excel (Microsoft) or SPSS (IBM). We compared independent sample means using *t*-tests and one-way ANOVAs as appropriate. Statistically significant *F*-values detected in the ANOVAs were followed by alpha-adjusted post-hoc tests (Tukey’s HSD). We confirmed necessary parametric test assumptions using the Shapiro–Wilk test (normality) and Levene’s test (error variance homogeneity). Violations in test assumption were corrected by transformations when possible; otherwise, the equivalent non-parametric tests were applied instead. Type-1 error rates for all tests were set at 0.05. For all bar graphs, center values represent mean, and error bars represent standard error of the mean (SEM).

### Reporting Summary

Further information on experimental design is available in the Nature Research Reporting Summary linked to this article.

## Supplementary information

Supplementary Information

Reporting Summary

## Data Availability

Raw proteomics data are available in the MassIVE database with the accession number MSV000083041. The rest of the datasets generated during and/or analyzed in the current study are available from the corresponding author on reasonable request.

## References

[1] Dailey ME, Smith SJ (1996). The dynamics of dendritic structure in developing hippocampal slices. J. Neurosci..

[2] Fiala JC, Feinberg M, Popov V, Harris KM (1998). Synaptogenesis via dendritic filopodia in developing hippocampal area CA1. J. Neurosci..

[3] Ziv NE, Smith SJ (1996). Evidence for a role of dendritic filopodia in synaptogenesis and spine formation. Neuron.

[4] Cooper MW, Smith SJ (1992). A real-time analysis of growth cone-target cell interactions during the formation of stable contacts between hippocampal neurons in culture. J. Neurobiol..

[5] Spence EF, Kanak DJ, Carlson BR, Soderling SH (2016). The Arp2/3 complex is essential for distinct stages of spine synapse maturation, including synapse unsilencing. J. Neurosci..

[6] Maletic-Savatic M, Malinow R, Svoboda K (1999). Rapid dendritic morphogenesis in CA1 hippocampal dendrites induced by synaptic activity. Science.

[7] Lendvai B, Stern EA, Chen B, Svoboda K (2000). Experience-dependent plasticity of dendritic spines in the developing rat barrel cortex in vivo. Nature.

[8] Lohmann C, Bonhoeffer T (2008). A role for local calcium signaling in rapid synaptic partner selection by dendritic filopodia. Neuron.

[9] Isaac JT, Nicoll RA, Malenka RC (1995). Evidence for silent synapses: implications for the expression of LTP. Neuron.

[10] Kim IH (2013). Disruption of Arp2/3 results in asymmetric structural plasticity of dendritic spines and progressive synaptic and behavioral abnormalities. J. Neurosci..

[11] Carlson BR (2011). WRP/srGAP3 facilitates the initiation of spine development by an inverse F-BAR domain, and its loss impairs long-term memory. J. Neurosci..

[12] Honkura N, Matsuzaki M, Noguchi J, Ellis-Davies GC, Kasai H (2008). The subspine organization of actin fibers regulates the structure and plasticity of dendritic spines. Neuron.

[13] Sigler A (2017). Formation and maintenance of functional spines in the absence of presynaptic glutamate release. Neuron.

[14] Kaufmann WE, Moser HW (2000). Dendritic anomalies in disorders associated with mental retardation. Cereb. Cortex.

[15] Fiala JC, Spacek J, Harris KM (2002). Dendritic spine pathology: cause or consequence of neurological disorders?. Brain Res. Brain Res. Rev..

[16] Ramakers GJ (2002). Rho proteins, mental retardation and the cellular basis of cognition. Trends Neurosci..

[17] Purpura DP (1974). Dendritic spine "dysgenesis" and mental retardation. Science.

[18] Uezu A (2016). Identification of an elaborate complex mediating postsynaptic inhibition. Science.

[19] Xu P, Zot AS, Zot HG (1995). Identification of Acan125 as a myosin-I-binding protein present with myosin-I on cellular organelles of Acanthamoeba. J. Biol. Chem..

[20] Jung G, Remmert K, Wu X, Volosky JM, Hammer JA (2001). The Dictyostelium CARMIL protein links capping protein and the Arp2/3 complex to type I myosins through their SH3 domains. J. Cell Biol..

[21] Yang C (2005). Mammalian CARMIL inhibits actin filament capping by capping protein. Dev. Cell.

[22] Liang Y, Niederstrasser H, Edwards M, Jackson CE, Cooper JA (2009). Distinct roles for CARMIL isoforms in cell migration. Mol. Biol. Cell.

[23] Akin O, Mullins RD (2008). Capping protein increases the rate of actin-based motility by promoting filament nucleation by the Arp2/3 complex. Cell.

[24] Iwasa JH, Mullins RD (2007). Spatial and temporal relationships between actin-filament nucleation, capping, and disassembly. Curr. Biol..

[25] Pollard TD, Borisy GG (2003). Cellular motility driven by assembly and disassembly of actin filaments. Cell.

[26] Suzuki K (2016). In vivo genome editing via CRISPR/Cas9 mediated homology-independent targeted integration. Nature.

[27] Monyer H, Burnashev N, Laurie DJ, Sakmann B, Seeburg PH (1994). Developmental and regional expression in the rat brain and functional properties of four NMDA receptors. Neuron.

[28] Frank RA (2016). NMDA receptors are selectively partitioned into complexes and supercomplexes during synapse maturation. Nat. Commun..

[29] Anderson GR (2017). Postsynaptic adhesion GPCR latrophilin-2 mediates target recognition in entorhinal-hippocampal synapse assembly. J. Cell Biol..

[30] Boucard AA, Maxeiner S, Sudhof TC (2014). Latrophilins function as heterophilic cell-adhesion molecules by binding to teneurins: regulation by alternative splicing. J. Biol. Chem..

[31] Um JW (2016). LRRTM3 regulates excitatory synapse development through alternative splicing and neurexin binding. Cell Rep..

[32] Cho KO, Hunt CA, Kennedy MB (1992). The rat brain postsynaptic density fraction contains a homolog of the Drosophila discs-large tumor suppressor protein. Neuron.

[33] Maynard KR, Stein E (2012). DSCAM contributes to dendrite arborization and spine formation in the developing cerebral cortex. J. Neurosci..

[34] Duman JG, Tu YK, Tolias KF (2016). Emerging roles of BAI adhesion-GPCRs in synapse development and plasticity. Neural Plast..

[35] Takahashi H, Craig AM (2013). Protein tyrosine phosphatases PTPdelta, PTPsigma, and LAR: presynaptic hubs for synapse organization. Trends Neurosci..

[36] Soderling SH (2007). A WAVE-1 and WRP signaling complex regulates spine density, synaptic plasticity, and memory. J. Neurosci..

[37] Fernandez E (2009). Targeted tandem affinity purification of PSD-95 recovers core postsynaptic complexes and schizophrenia susceptibility proteins. Mol. Syst. Biol..

[38] Bayes A (2012). Comparative study of human and mouse postsynaptic proteomes finds high compositional conservation and abundance differences for key synaptic proteins. PLoS ONE.

[39] Collins MO (2006). Molecular characterization and comparison of the components and multiprotein complexes in the postsynaptic proteome. J. Neurochem..

[40] Huang da W, Sherman BT, Lempicki RA (2009). Systematic and integrative analysis of large gene lists using DAVID bioinformatics resources. Nat. Protoc..

[41] Edwards M, Liang Y, Kim T, Cooper JA (2013). Physiological role of the interaction between CARMIL1 and capping protein. Mol. Biol. Cell.

[42] Viswanathan S (2015). High-performance probes for light and electron microscopy. Nat. Methods.

[43] Miesenbock G, De Angelis DA, Rothman JE (1998). Visualizing secretion and synaptic transmission with pH-sensitive green fluorescent proteins. Nature.

[44] Fujiwara I, Remmert K, Piszczek G, Hammer JA (2014). Capping protein regulatory cycle driven by CARMIL and V-1 may promote actin network assembly at protruding edges. Proc. Natl. Acad. Sci. USA.

[45] Fan Y, Tang X, Vitriol E, Chen G, Zheng JQ (2011). Actin capping protein is required for dendritic spine development and synapse formation. J. Neurosci..

[46] McClatchy DB, Liao L, Lee JH, Park SK, Yates JR (2012). Dynamics of subcellular proteomes during brain development. J. Proteome Res..

[47] Gonzalez-Lozano MA (2016). Dynamics of the mouse brain cortical synaptic proteome during postnatal brain development. Sci. Rep..

[48] Huttenlocher PR, Dabholkar AS (1997). Regional differences in synaptogenesis in human cerebral cortex. J. Comp. Neurol..

[49] Petanjek Z (2011). Extraordinary neoteny of synaptic spines in the human prefrontal cortex. Proc. Natl. Acad. Sci. USA.

[50] Darnell JC (2001). Fragile X mental retardation protein targets G quartet mRNAs important for neuronal function. Cell.

[51] Li J (2014). Integrated systems analysis reveals a molecular network underlying autism spectrum disorders. Mol. Syst. Biol..

[52] Iossifov I (2015). Low load for disruptive mutations in autism genes and their biased transmission. Proc. Natl. Acad. Sci. USA.

[53] Houge G, Rasmussen IH, Hovland R (2012). Loss-of-function CNKSR2 mutation is a likely cause of non-syndromic X-linked intellectual disability. Mol. Syndromol..

[54] McRae JF (2017). Prevalence and architecture of de novo mutations in developmental disorders. Nature.

[55] Endris V (2002). The novel Rho-GTPase activating gene MEGAP/ srGAP3 has a putative role in severe mental retardation. Proc. Natl. Acad. Sci. USA.

[56] Soderling SH (2002). The WRP component of the WAVE-1 complex attenuates Rac-mediated signalling. Nat. Cell Biol..

[57] Endris V (2011). SrGAP3 interacts with lamellipodin at the cell membrane and regulates Rac-dependent cellular protrusions. J. Cell. Sci..

[58] Lim J, Ritt DA, Zhou M, Morrison DK (2014). The CNK2 scaffold interacts with vilse and modulates Rac cycling during spine morphogenesis in hippocampal neurons. Curr. Biol..

[59] Kim T, Ravilious GE, Sept D, Cooper JA (2012). Mechanism for CARMIL protein inhibition of heterodimeric actin-capping protein. J. Biol. Chem..

[60] Zwolak A, Fujiwara I, Hammer JA, Tjandra N (2010). Structural basis for capping protein sequestration by myotrophin (V-1). J. Biol. Chem..

[61] Uruno T, Remmert K, Hammer JA (2006). CARMIL is a potent capping protein antagonist: identification of a conserved CARMIL domain that inhibits the activity of capping protein and uncaps capped actin filaments. J. Biol. Chem..

[62] Takeda S (2010). Two distinct mechanisms for actin capping protein regulation—steric and allosteric inhibition. PLoS Biol..

[63] Zwolak A, Uruno T, Piszczek G, Hammer JA, Tjandra N (2010). Molecular basis for barbed end uncapping by CARMIL homology domain 3 of mouse CARMIL-1. J. Biol. Chem..

[64] Schwenk F, Baron U, Rajewsky K (1995). A cre-transgenic mouse strain for the ubiquitous deletion of loxP-flanked gene segments including deletion in germ cells. Nucleic Acids Res..

[65] Ran FA (2013). Genome engineering using the CRISPR-Cas9 system. Nat. Protoc..

[66] Swiech L (2015). In vivo interrogation of gene function in the mammalian brain using CRISPR-Cas9. Nat. Biotechnol..

[67] Shaner NC (2004). Improved monomeric red, orange and yellow fluorescent proteins derived from Discosoma sp. red fluorescent protein. Nat. Biotechnol..

