# Supplementary material for: In vivo proximity proteomics of nascent synapses reveals a novel regulator of cytoskeleton-mediated synaptic maturation
Source: Nat Commun. 2019 Jan 23;10:386. doi: 10.1038/s41467-019-08288-w (PMC6344529; doi:10.1038/s41467-019-08288-w)
Supplement: Supplementary file 1 — Supplementary Information [file 41467_2019_8288_MOESM1_ESM.pdf]

Supplementary Information

In vivo proximity proteomics of nascent synapses reveals a novel regulator of cytoskeleton-mediated synaptic maturation

Spence and Dube *et al.*

Contents

Supplementary Figures 1 – 10

Supplementary Tables 1 – 2

Supplementary References

a

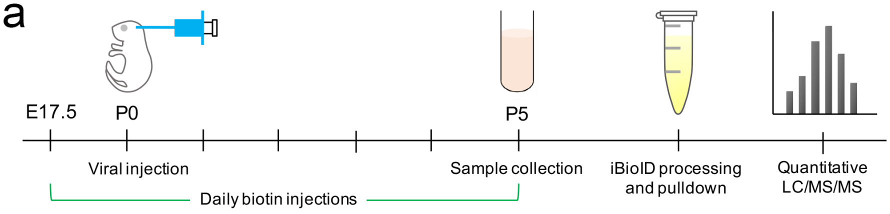

b

BioID Probes	Biotinylated Samples
Wrp-BirA	Biotinylation by dendritic-filopodial targeted BirA
MT-BirA	Biotinylation by membrane-targeted BirA
BirA	Biotinylation by non-targeted BirA

c

		Wrp-BirA	MT-BirA
	Number of unique proteins	2,490	
Filter 1	Removal of carboxylase and keratin contaminants	2,463	
Filter 2	Proteins with 2 or more peptides identified	1,643	
Filter 3	Proteins enriched more than 2.5 fold over BirA control	106	183
Filter 4	Proteins known to be contaminants or reside at other subcellular sites (secreted, mitochondrial)	88	177
Filter 5	Proteins with p-value <0.05	60	117

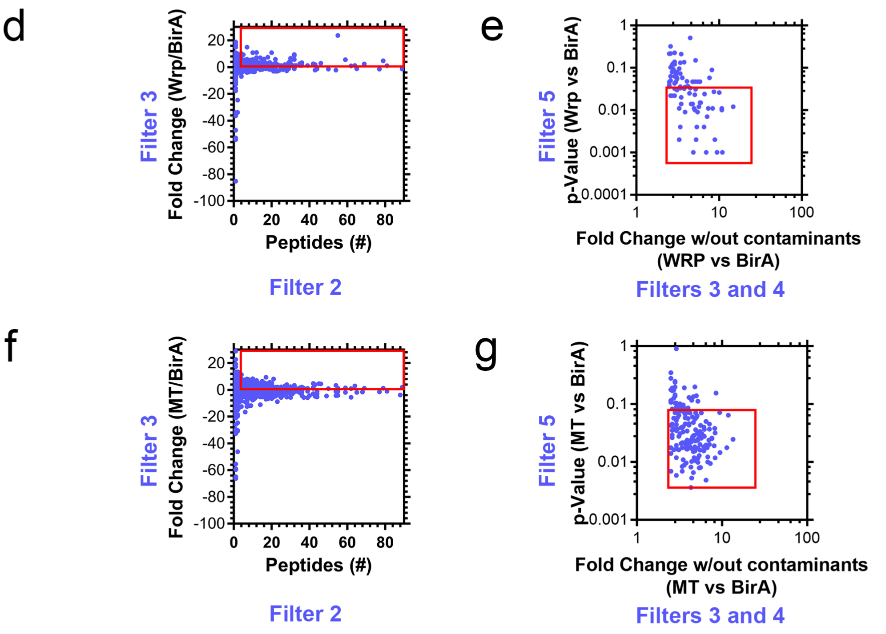

Supplementary Figure 1. ***Layout of proteomic study design and designated filters.*** **a,** Schematic of timeline for developmental iBioID. **b,** Chart summarizing probe biotinylation targets. **c,** Chart summarizing proteomic dataset identified by mass spectrometry and filters used to identify top candidates. **d,** Graphical representation of filters 2 and 3 from Wrp-BirA data set. **e,** Graphical representation of filters 3, 4, and 5 from Wrp-BirA data set. **f,** Graphical representation of filters 2 and 3 from MT-BirA data set. **g,** Graphical representation of filters 3, 4, and 5 from the MT-BirA data set.

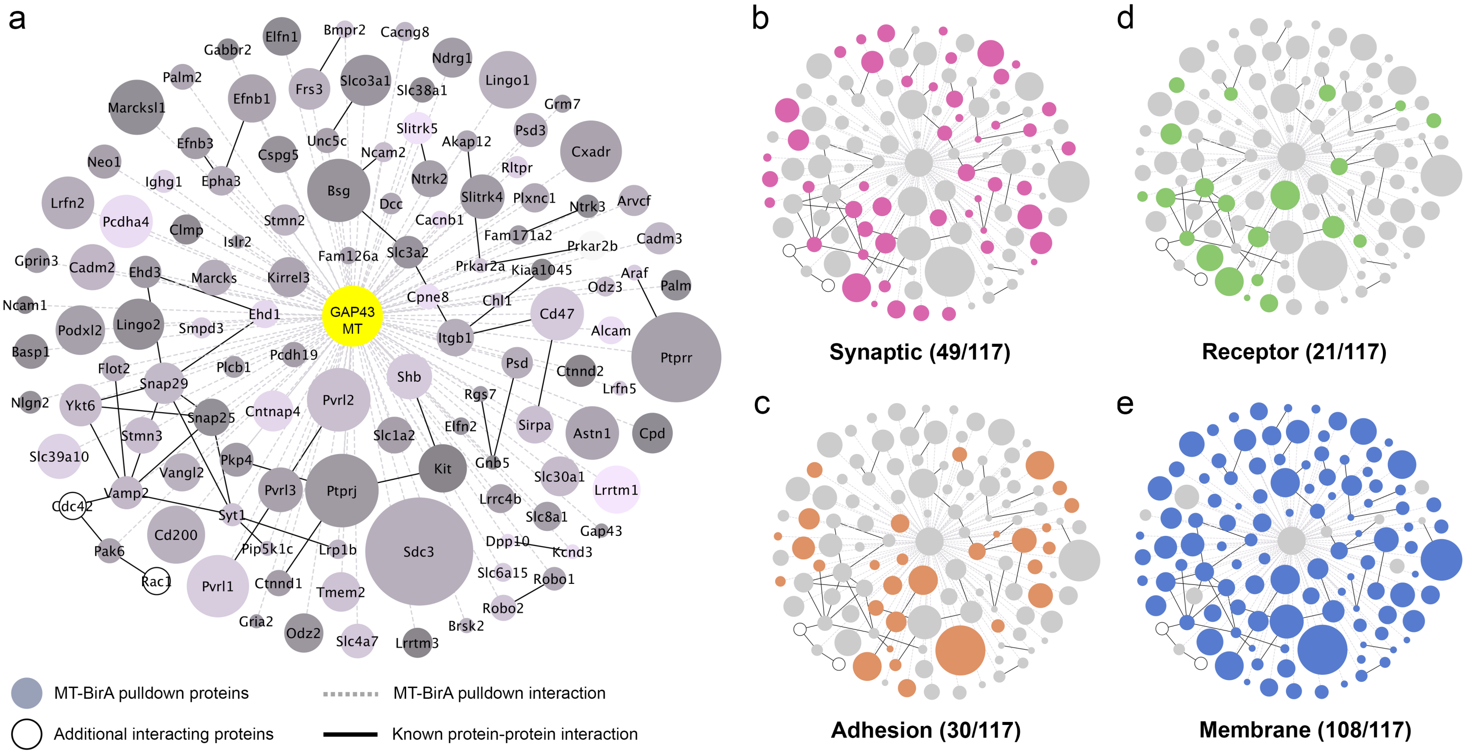

Supplementary Figure 2. ***Proteome of the membranes of developing neurons.*** **a,** MT-BirA dependent iBioID identified a network of known and unknown proteins enriched at neuronal membranes during the first postnatal week of development. Node titles correspond to the gene name, size represents fold enrichment over the BirA negative control (range 2.47 – 13.54), shading represents p-value with light gray being a lower p-value and darker gray a higher p-value (range 0.0036 – 0.05). Edges are marked according to type of interaction. Dashed lines are iBioID interaction where as solid lines are previously known interactions identified in the Genemania, Biogrid, or Sting databases. **b,** Clustergram of synaptic proteins (pink) as identified through DAVID analysis or the G2C database of Cortex PSD Consensus proteins[1]. **c,** Clustergram of adhesion proteins (orange) as identified through DAVID analysis. **d,** Clustergram of receptor proteins (green) as identified through DAVID analysis. **e,** Clustergram of membrane proteins (blue) as identified through DAVID analysis.

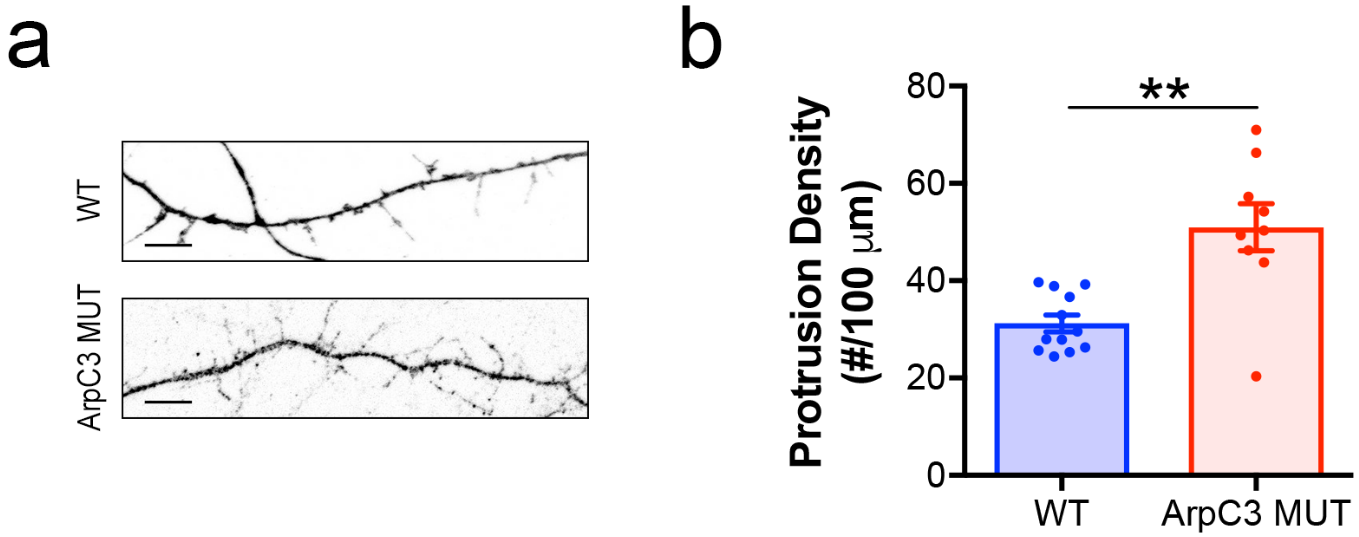

Supplementary Figure 3. ***Virally mediated Cas9 depletion of ArpC3.*** **a,** Representative images of control (WT) and ArpC3-depleted (mutant, MUT) neurons. Scale bars are 5 μm. **b,** Graphical representation of dendritic protrusions for WT (31 ± 2 protrusions, n = 12 neurons) and MUT (51 ± 5 protrusions, n = 9 neurons). p = 0.0004. **p<0.01, t-tests. Error bars are standard error of the mean (SEM).

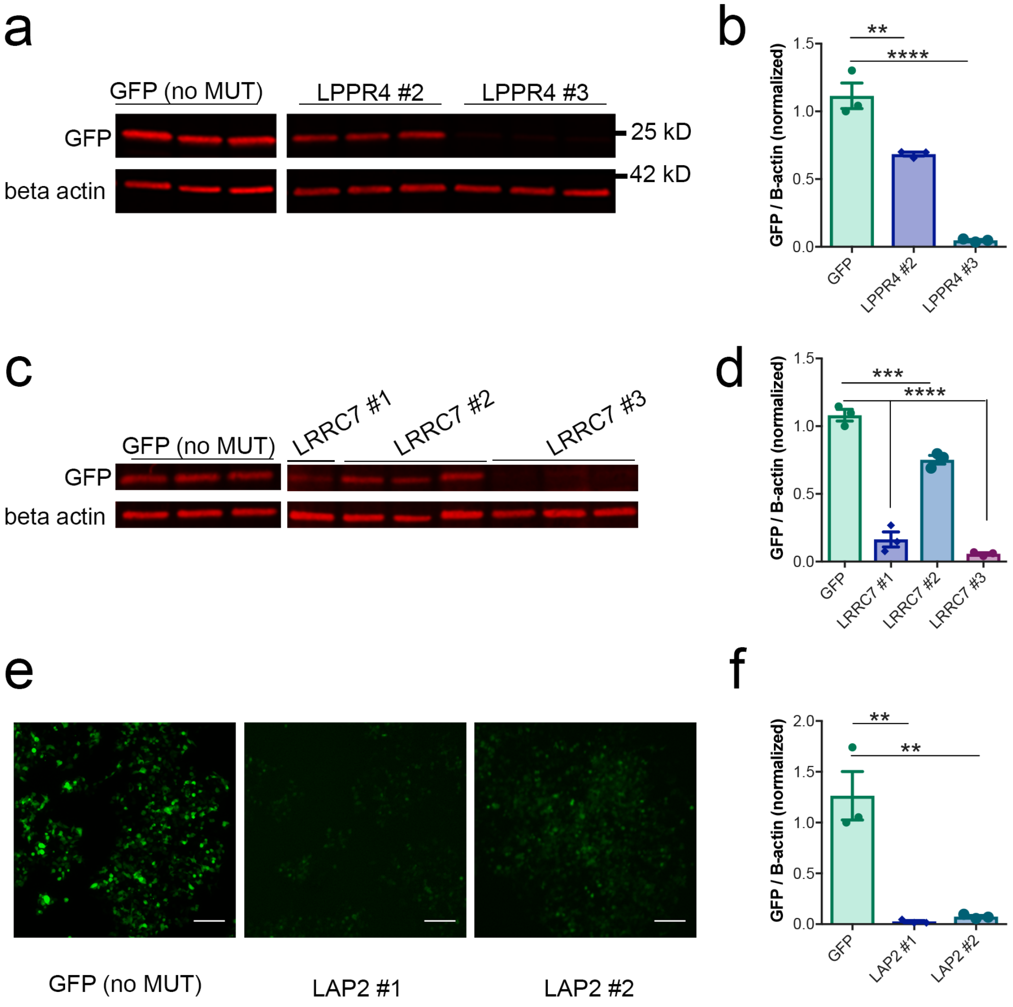

Supplementary Figure 4. ***Guide validation of LPPR4, LRRC7, and LAP2 guides.*** **a,** Representative blot of LPPR4 knockdown of GFP with LPPR4 guides 2 and 3. **b,** Graphical representation of GFP intensity for Control (1.114 ± 0.094 a.u., n = 3 transfections), LPPR4 guide 2 (0.685 ± 0.015 a.u., n = 3 transfections) and LPPR4 guide 3 (0.048 ± 0.007 a.u., n= 3 transfections). $F_{2,6}$= 94.37, $p<0.0001$. **c,** Representative blot of LPPR7 knockdown of GFP with LRRC7 guides 1, 2, and 3. **d,** Graphical representation of GFP intensity for Control (1.081 ± 0.043 a.u., n = 3 transfections), LRRC7 guide 1 (0.164 ± 0.056 a.u., n = 3 transfections), LRRC7 guide 2 (0.752 ± 0.032 a.u., n= 3 transfections), and LRRC7 guide 3 (0.058 ± 0.008 a.u., n = 3 transfections). $F_{3,8}$= 156.5, $p<0.0001$. **e,** Representative images of HEK293T cells transfected with GFP with and without guides against LAP2. Scale bars are 10 mm. **f,** Graphical representation of GFP intensity for Control (1.265 ± 0.239 a.u., n = 3 transfections), LAP2 guide 1 (0.025 ± 0.011 a.u., n = 3 transfections) and LAP2 guide 2 (0.07 ± 0.012 a.u., n= 3 transfections). $F_{2,6}$= 25.85, p= 0.0011. **p<0.01, ***p<0.001, ****p<0.0001, one-way ANOVAs. Error bars are standard error of the mean (SEM).

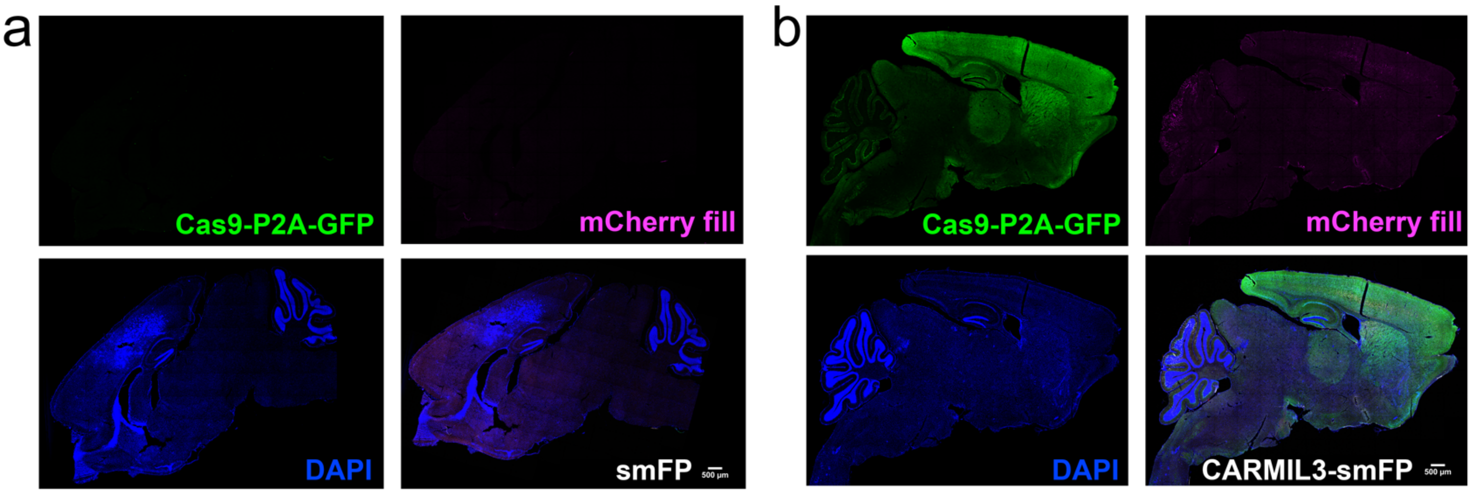

Supplementary Figure 5. ***Validation of HITI KI CARMIL3 expression in whole mouse brain.*** **a,** Control (uninjected) P14 mouse brain cryosection. **b,** P14 mouse brain cryosection from pup injected with AAV-U6-CARMIL3Ctermsg1-HITI-smFP-P2A-mCherry-SynI-Cre. Green is Cas9-P2A-GFP reporter, magenta is mCherry, blue is DAPI, and white is CARMIL3-smFP. Scale bars are 500 μm.

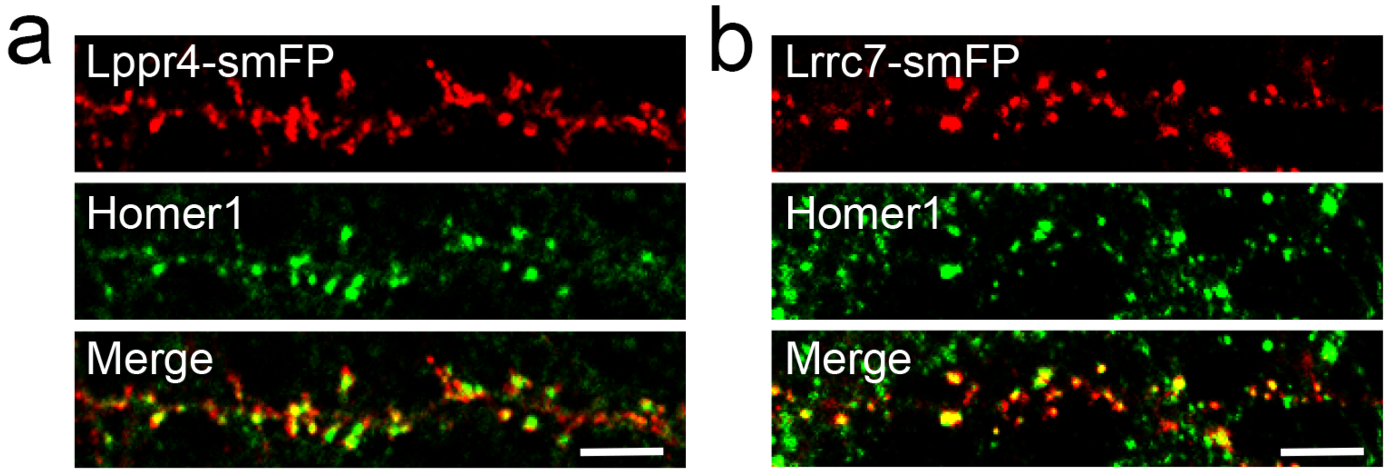

Supplementary Figure 6. ***Early synaptic candidate proteins, Lppr4 and Lrrc7 are expressed in hippocampal neurons and co-localize with the excitatory postsynaptic marker Homer1.*** Cultured hippocampal neurons from Cas9 KI P0 pups were transduced with AAV-HITI-smFP to label endogenous **(a)** Lppr4 or **(b)** Lrrc7 using specific sgRNAs targeting C terminus of each genes. At DIV14, cells were co-stained with Homer1 as an excitatory postsynaptic marker. Red- smFP, green- Homer1, white- Merge. Scale bars are 5 μm.

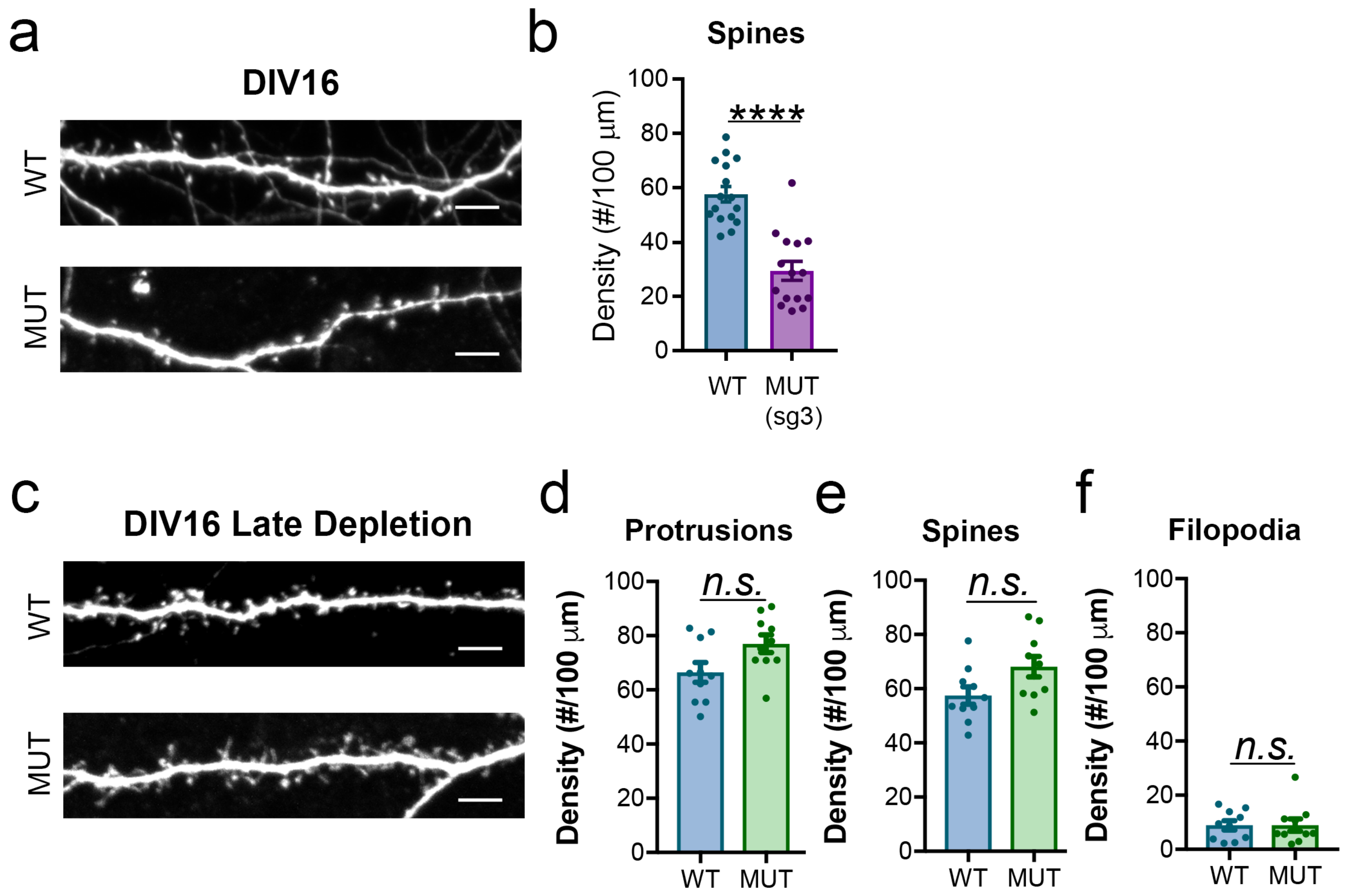

Supplementary Figure 7. ***CARMIL3 depletion with a second guide, sg3, reveals consistent changes with morphological maturation.*** **a,** Representative images of WT and MUT neurons (using CARMIL3 sg 3) at DIV16. **b,** Graphical representation of dendritic spine density for WT (58 ± 3 protrusions, n = 16 neurons) and MUT (29 ± 3 protrusions, n = 15 neurons) neurons at DIV16. p<0.0001. **c,** Representative images of WT and late MUT neurons at DIV14. **d,** Graphical representation of dendritic protrusion density for WT (66 ± 4 protrusions, n = 10 neurons) and late MUT (77 ± 3 protrusions, n = 10 neurons) neurons at DIV16. p=0.172. **e,** Graphical representation of dendritic spine density for WT (58 ± 3 spines, n = 10 neurons) and late MUT (68 ± 4 spines, n = 10 neurons) neurons at DIV16. p=0.226. **f,** Graphical representation of dendritic filopodia density for WT (9 ± 2 filopodia, n = 10 neurons) and late MUT (9 ± 2 filopodia, n = 10 neurons) neurons at DIV16. p=0.832. ****p<0.0001, t-tests. Scale bars, 5 μm. Error bars are standard error of the mean (SEM).

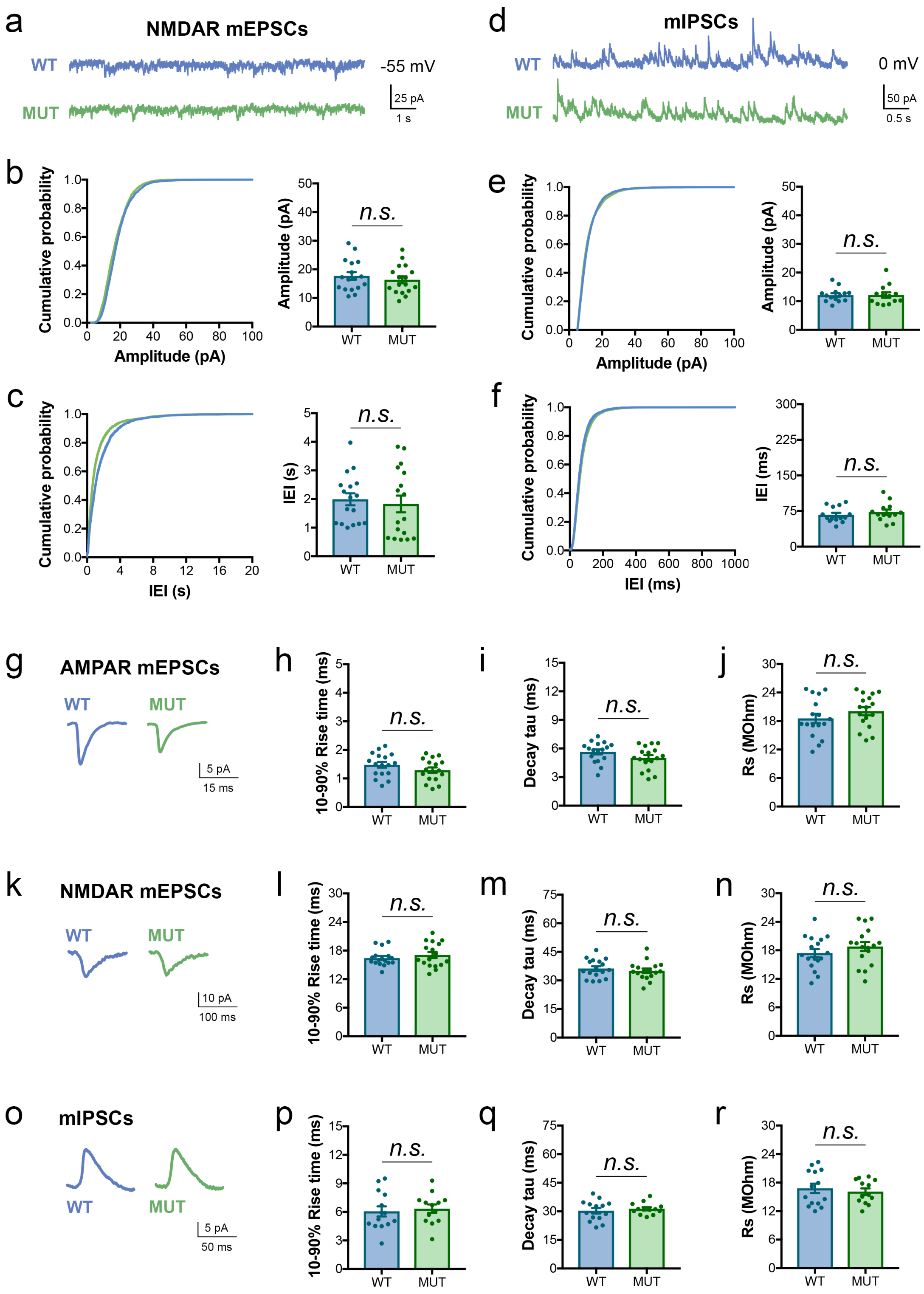

Supplementary Figure 8. ***CARMIL3 depletion does not affect NMDAR- or GABAR- mediated currents, nor does it affect kinetics of any postsynaptic currents.*** **a,** Representative traces of pharmacologically isolated NMDAR-mediated mEPSCs recorded from WT and MUT neurons at DIV12-16. **b,** Amplitude cumulative probability plots for NMDAR-mEPSCs. Quantification on the right shows average NMDAR-mEPSC amplitude for WT (17.70 ± 1.33 pA, n = 17 neurons) and MUT (16.37 ± 1.21 pA, n = 17 neurons) neurons. p=0.4643. **c,** IEI

cumulative probability plots for NMDAR-mEPSCs. Quantification on the right shows average NMDAR-mEPSC IEI for WT (1.99 ± 0.21 s, n = 17 neurons) and MUT (1.83 ± 0.29 s, n = 17 neurons) neurons. p=0.6528. **d,** Representative traces of pharmacologically isolated mIPSCs recorded from WT and MUT neurons at DIV12-16. **e,** Amplitude cumulative probability plots for mIPSCs. Quantification on the right shows average mIPSC amplitude for WT (12.13 ± 0.64 pA, n = 14 neurons) and MUT (12.14 ± 0.96 pA, n = 13 neurons) neurons. p=0.9942. **f,** IEI cumulative probability plots for mIPSCs. Quantification on the right shows average mIPSC IEI for WT (67.07 ± 4.26 ms, n = 14 neurons) and MUT (72.69 ± 5.45 ms, n = 13 neurons) neurons. p=0.4250. **g,** Representative waveform average of pharmacologically isolated AMPAR-mediated mEPSCs recorded from control (WT, blue) and CARMIL3-depleted (MUT, green) neurons at DIV12-16. **h,** Graphical representation of AMPAR-mEPSC 10-90% rise time for WT (1.48 ± 0.10 ms, n = 17 neurons) and MUT (1.29 ± 0.10 ms, n = 17 neurons) neurons. p=0.1860. **i,** Graphical representation of AMPAR-mEPSC decay time constant for WT (5.65 ± 0.26 ms, n = 17 neurons) and MUT (5.01 ± 0.30 ms, n = 17 neurons) neurons. p=0.1168. **j,** Graphical representation of series resistance for WT (18.54 ± 1.00 MΩ, n = 17 neurons) and MUT (20.06 ± 0.85 MΩ, n = 17 neurons) neurons during AMPAR-mEPSC recordings. p=0.2552. **k,** Representative waveform average of pharmacologically isolated NMDAR-mediated mEPSCs recorded from WT and MUT neurons at DIV12-16. **l,** Graphical representation of NMDAR-mEPSC 10-90% rise time for WT (16.43 ± 0.41 ms, n = 17 neurons) and MUT (17.09 ± 0.62 ms, n = 17 neurons) neurons. p=0.3905. **m,** Graphical representation of NMDAR-mEPSC decay time constant for WT (36.22 ± 1.19 ms, n = 17 neurons) and MUT (35.06 ± 1.16 ms, n = 17 neurons) neurons. p=0.4912. **n,** Graphical representation of series resistance for WT (17.43 ± 0.84 MΩ, n = 17 neurons) and MUT (18.81 ± 0.95 MΩ, n = 17 neurons) neurons during NMDAR-mEPSC recordings. p=0.2849. **o,** Representative waveform average of pharmacologically isolated mIPSCs recorded from WT and MUT neurons at DIV12-16. **p,** Graphical representation of mIPSC 10-90% rise time for WT (6.06 ± 0.53 ms, n = 14 neurons) and MUT (6.34 ± 0.45 ms, n = 13 neurons) neurons. p=0.6873. **q,** Graphical representation of mIPSC decay time constant for WT (30.31 ± 1.46 ms, n = 14 neurons) and MUT (31.34 ± 0.85 ms, n = 13 neurons) neurons. p=0.5472. **r,** Graphical representation of series resistance for WT (16.79 ± 1.00 MΩ, n = 14 neurons) and MUT (16.09 ± 0.69 MΩ, n = 13 neurons) neurons during mIPSC recordings. p=0.5698, t-tests. Error bars are standard error of the mean (SEM).

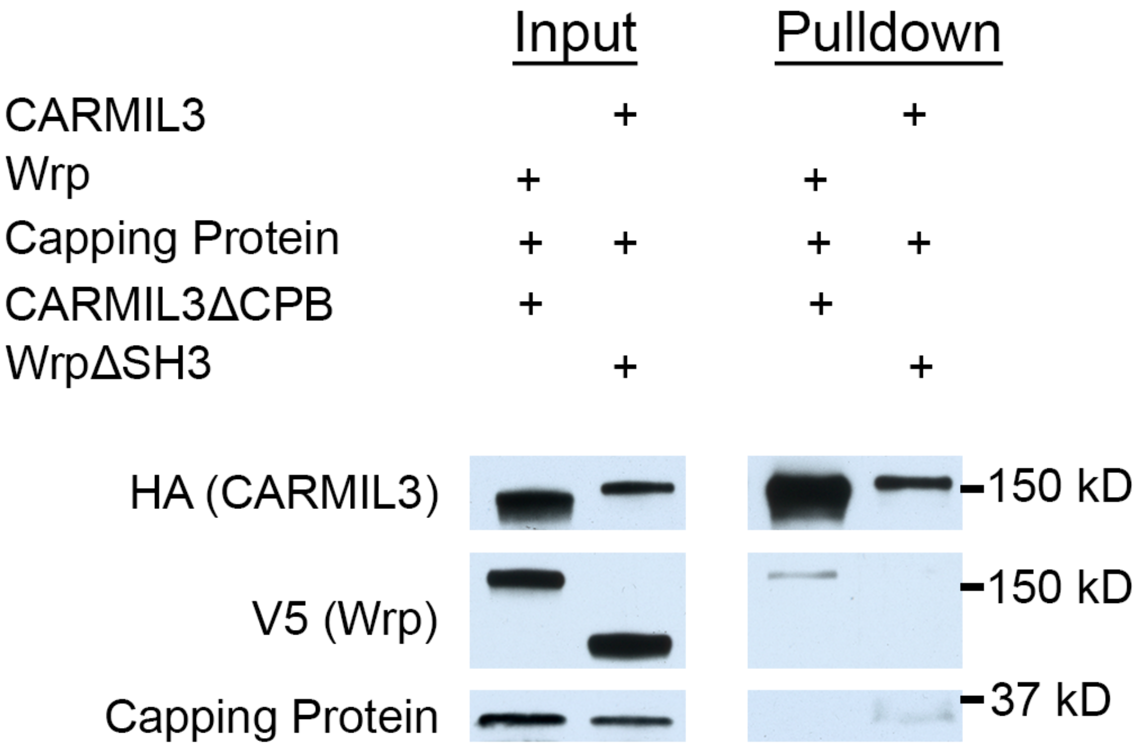

Supplementary Figure 9. ***CARMIL3 interacts with Capping Protein and Wrp through distinctive domains.*** Pulldowns of HA epitope tagged CARMIL3 or CARMIL3 with a deletion of the CP binding motif. CARMIL3, Wrp, and CP constructs were overexpressed in HEK293T cells.

Figure 3b, left panel

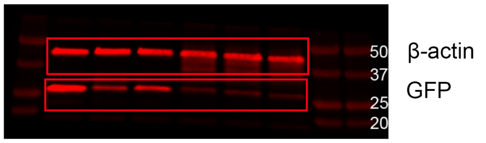

Figure 3b, right panel

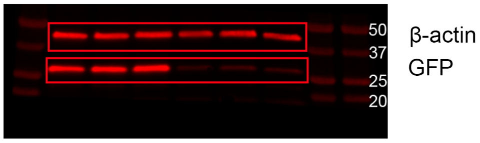

Figure 4b

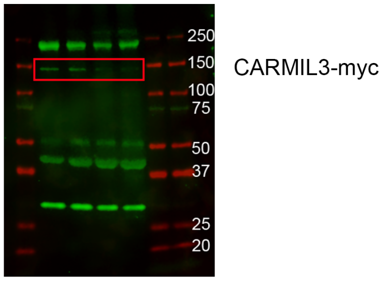

Figure 7b

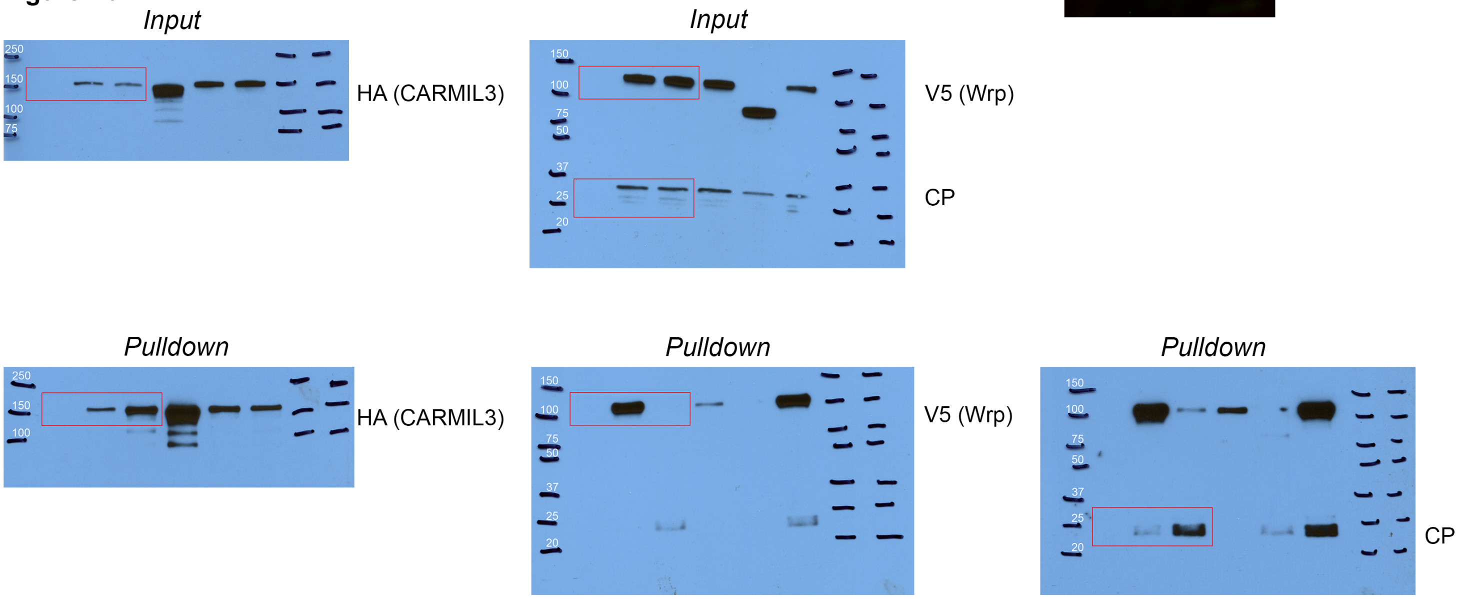

Figure 7c

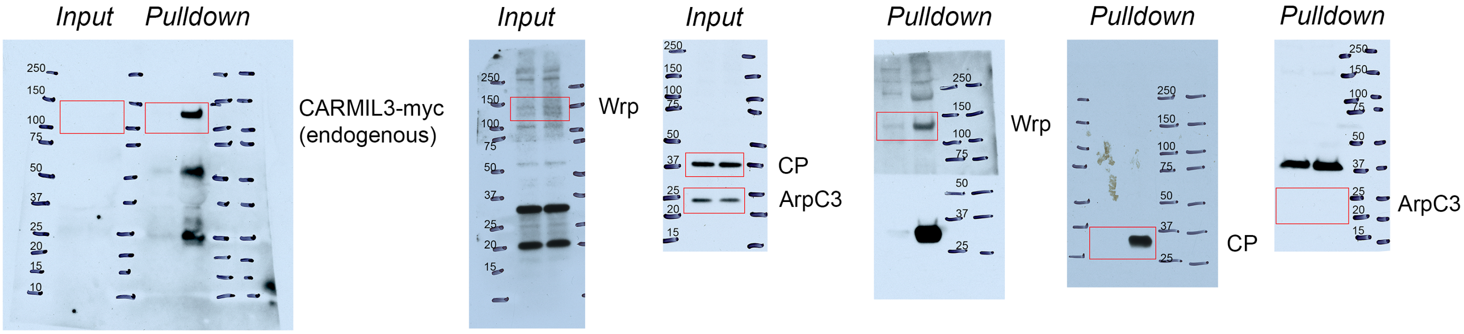

Supplementary Figure 10. *Original blots.* Uncropped scans of Western blots used in the current study.

Supplementary Table 1. *Expression constructs details.* A detailed list of constructs included in the paper.

Construct	Promoter	Insert(s)	Source	Figure(s)
pAAV-BirA-HA	Synapsin	BirA-HA	generated in Soderling Lab[2]	Figure 1
pAAV-MT-BirA-HA	Synapsin	Membrane Tag (Gap43)-BirA-HA	generated from pAAV-BirA-HA	Figure 1
pAAV-Wrp (IFBAR)-BirA-HA	Synapsin	Wrp (IFBAR)-BirA-HA	generated from pAAV-BirA-HA	Figure 1
pBA-tdTomato	β-actin	tdTomato	generated in Soderling Lab	Figure 1
PX458	U6, CBh	empty sgRNA, SpCas9-P2A-GFP	pSpCas9(BB)-P2A-GFP (PX458) was a gift from Feng Zhang (Addgene plasmid # 48138)	Figure 3
pAAV-U6-empty sgRNA-hSynI-Cre	U6, Synapsin	empty sgRNA, Cre	generated from PX458	Figure 3, Figure 4S, Figure 5, Figure 6, Figure S7
pAAV-U6-ArpC3 sg1-hSynI-Cre	U6	TCCTTTGAACTGGCTTCGGA	generated from pAAV-U6-empty sgRNA-hSynI-Cre	Figure 3
pAAV-U6-ArpC3 sg2-hSynI-Cre	U6	GTCCTTTGAACTGGCTTCGG	generated from pAAV-U6-empty sgRNA-hSynI-Cre	Figure 3
pAAV-U6-CARMIL3 sg1-hSynI-Cre	U6	CTCGCGGGTGAGCTCCACGC	generated from pAAV-U6-empty sgRNA-hSynI-Cre	Figure 3
pAAV-U6-CARMIL3 sg2-hSynI-Cre	U6	CCAGCAACATCGTGTGAAAC	generated from pAAV-U6-empty sgRNA-hSynI-Cre	Figure 3
pAAV-U6-CARMIL3 sg3-hSynI-Cre	U6	CACTCTGTCTTCGAACTTCT	generated from pAAV-U6-empty sgRNA-hSynI-Cre	Figure 3, Figure S7
pBA-GFP	β-actin	GFP	generated in Soderling Lab	Figure 3, Figure 4, Figure S4
pBA-ArpC3 sg1 and sg2 target-GFP	β-actin	atggcgCCCTCCGAAGCCAGTTCAAAGGAC-GFP	generated from pBA-GFP	Figure 3
pBA-CARMIL3 sg1 target-GFP	β-actin	atggcgCCAGCGTGGAGCTCACCCGCGAGc-GFP	generated from pBA-GFP	Figure 3
pBA-CARMIL3 sg2 target-GFP	β-actin	atggcgCCAGCAACATCGTGTGAAACTGGc-GFP	generated from pBA-GFP	Figure 3
pBA-CARMIL3 sg3 target-GFP	β-actin	atggcgCCAAGAAGTTCGAAGACAGAGTGctgc-GFP	generated from pBA-GFP	Figure 3
pAAV-FLEx-tdTomato	CAG	FLEx-tdTomato	pAAV-FLEx-tdTomato was a gift from Edward Boyden (Addgene plasmid # 28306)	Figure 3, Figure 5, Figure 6, Figure S7, Figure 7
PX330	CBh	hSpCas9	pX330-U6-Chimeric_BB-CBh-hSpCas9 was a gift from Feng Zhang (Addgene plasmid # 42230)	Figure 3, Figure 4
pAAV-U6-CARMIL3 Cterm sg1-HITI-myc-P2A-mCherry-hSynI-Cre	U6, Synapsin	TCAGTCTGTTCCTGGTTCAC, Cre, HITI-myc-P2A-mCherry	generated from pAAV-U6-empty sgRNA-hSynI-Cre	Figure 4
pAAV-U6-CARMIL3 Cterm sg1-HITI-smFP-P2A-mCherry-hSynI-Cre	U6, Synapsin	TCAGTCTGTTCCTGGTTCAC, Cre, HITI-smFP-P2A-mCherry	generated from pAAV-U6-empty sgRNA-hSynI-Cre	Figure 4

pAAV-Cas9 (PX551)	Mecp2	SpCas9	PX551 was a gift from Feng Zhang (Addgene plasmid # 60957)	Figure 4, Figure 7
pAAV-U6-CARMIL3 sg1-HITI-P2A-Cre-STOP	U6	CTCGCGGGTGAGCTCCACGC, P2A-Cre-STOP	generated from pAAV-U6-empty sgRNA-hSynI-Cre	Figure 5, Figure 6, Figure S7
pBA-SEP-GluA1	β-actin	SEP-GluA1	generated in Soderling Lab	Figure 6
pBA-SEP-GluA2	β-actin	SEP-GluA2	generated in Soderling Lab	Figure 6
pBA-HA-CARMIL3	β-actin	HA-CARMIL3	generated in Soderling Lab	Figure 7, Figure S8
pEF1α-Wrp	EF1α	Wrp	generated in Soderling Lab	Figure 7, Figure S8
pBA-CARMIL3 ΔWrpB	β-actin	CARMIL3 ΔWrpB	generated in Soderling Lab	Figure 7
pCMV-CAPZB2-TwinStrepII	CMV	CAPZB2-TwinStrepII	CAPZB2-TwinStrepII was a gift from Peter Barr-Gillespie (Addgene plasmid # 83194)	Figure 7, Figure S8
pAAV-U6-LPPR4 sg2-hSynI-Cre	U6	GGCGGCCGGGATGTCGGCGA	generated from pAAV-U6-empty sgRNA-hSynI-Cre	Figure S4
pAAV-U6-LPPR4 sg3-hSynI-Cre	U6	TGGTTCCAGCGGTGCCCGCG	generated from pAAV-U6-empty sgRNA-hSynI-Cre	Figure S4
pAAV-U6-LRRC7 sg1-hSynI-Cre	U6	ATGCCGCTGCTTCCGTGGAG	generated from pAAV-U6-empty sgRNA-hSynI-Cre	Figure S4
pAAV-U6-LRRC7 sg2-hSynI-Cre	U6	CACACTAGAAGAGCTCTATC	generated from pAAV-U6-empty sgRNA-hSynI-Cre	Figure S4
pAAV-U6-LRRC7 sg3-hSynI-Cre	U6	TTCGAAGTTGAAGACCTCCT	generated from pAAV-U6-empty sgRNA-hSynI-Cre	Figure S4
pAAV-U6-LAP2 sg1-hSynI-Cre	U6	GTACCATGTCGCTGTCTACG	generated from pAAV-U6-empty sgRNA-hSynI-Cre	Figure S4
pAAV-U6-LAP2 sg2-hSynI-Cre	U6	GACGAAGTTTGTTTGTGCGGT	generated from pAAV-U6-empty sgRNA-hSynI-Cre	Figure S4
pBA-LPPR4 sg2 target-GFP	β-actin	atggcgAATGTGACATCAGCGGCGCCGGGc-GFP	generated from pBA-GFP	Figure S4
pBA-LPPR4 sg3 target-GFP	β-actin	atggcgTGGTTCCAGCGGTGCCCGCGGGGc-GFP	generated from pBA-GFP	Figure S4
pBA-LRRC7 sg1 target-GFP	β-actin	atggcgATGCCGCTGCTTCCGTGGAGAGGca-GFP	generated from pBA-GFP	Figure S4
pBA-LRRC7 sg2 target-GFP	β-actin	atggcgCACACTAGAAGAGCTCTATCTGGc-GFP	generated from pBA-GFP	Figure S4
pBA-LRRC7 sg3 target-GFP	β-actin	atggcgTTCGAAGTTGAAGACCTCCTTGGc-GFP	generated from pBA-GFP	Figure S4
pBA-LAP2 sg1 target-GFP	β-actin	atggcgaccGTACCATGTCGCTGTCTACGAGGc-GFP	generated from pBA-GFP	Figure S4
pBA-LAP2 sg2 target-GFP	β-actin	atggcgaccACGAAGTTTGTTTGTGCGGTTGGc-GFP	generated from pBA-GFP	Figure S4
pAAV-U6-LPPR4 Cterm sg1-HITI-smFP	U6	CAAAGGAACCTCCCCCACGC	generated from pAAV-U6-empty sgRNA-hSynI-Cre	Figure S6
pAAV-U6-LRRC7 Cterm sg1-HITI-smFP	U6	TGAGCTCACGTTGAATAACT	generated from pAAV-U6-empty sgRNA-hSynI-Cre	Figure S6
pBA-CARMIL3 ΔCPB	β-actin	CARMIL3 ΔCPB	generated in Soderling Lab	Figure S8
pEF1α-Wrp ΔSH3	EF1α	Wrp ΔSH3	generated in Soderling Lab	Figure S8

Supplementary Table 2. ***AAV virus details.*** A detailed list of viruses included in the paper.

Virus	Serotype	Construct	Producer	Figure(s)
AAV-BirA-HA	9	pAAV-BirA-HA	University of Pennsylvania Viral Core	Figure 2, Figure S2
AAV-MT-BirA-HA	9	pAAV-MembraneTag-BirA-HA	University of Pennsylvania Viral Core	Figure S2
AAV-Wrp-BirA-HA	9	pAAV-Wrp(IFBAR)-BirA-HA	University of Pennsylvania Viral Core	Figure 2
AAV-U6-empty sgRNA-hSynI-Cre	2/9	pAAV-U6-empty sgRNA-hSynI-Cre	Soderling Lab	Figure 3, Figure S3, Figure 5, Figure 6, Figure S7, Figure 7
AAV-U6-CARMIL3 sg1-hSynI-Cre	2/9	pAAV-U6-CARMIL3 sg1-hSynI-Cre	Soderling Lab	Figure 3, Figure 5
AAV-U6-LPPR4sg3-hSynI-Cre	2/9	pAAV-U6-LPPR4 sg3-hSynI-Cre	Soderling Lab	Figure 3
AAV-U6-LRRC7sg3-hSynI-Cre	2/9	pAAV-U6-LRRC7 sg3-hSynI-Cre	Soderling Lab	Figure 3
AAV-U6-LAP2sg1-hSynI-Cre	2/9	pAAV-U6-LAP2 sg1-hSynI-Cre	Soderling Lab	Figure 3
AAV-FLEx-tdTomato	9	pAAV-FLEx-tdTomato	University of Pennsylvania Viral Core	Figure 3, Figure 5, Figure 6, Figure S7, Figure 7
AAV-U6-CARMIL3 Cterm sg1-HITI-myc-P2A-mCherry-hSynI-Cre	2/9	pAAV-U6-CARMIL3 Cterm sg1-HITI-myc-P2A-mCherry-hSynI-Cre	Soderling Lab	Figure 4, Figure 7
AAV-U6-CARMIL3 Cterm sg1-HITI-smFP-P2A-mCherry-hSynI-Cre	2/9	pAAV-U6-CARMIL3 Cterm sg1-HITI-smFP-P2A-mCherry-hSynI-Cre	Soderling Lab	Figure 4
AAV-Cas9	2/9	PX551	Soderling Lab	Figure 4, Figure 7
AAV-U6-CARMIL3 sg1-HITI-P2A-Cre-STOP	2/9	pAAV-U6-CARMIL3 sg1-HITI-P2A-Cre-STOP	Soderling Lab	Figure 5, Figure 6, Figure S7, Figure 7
AAV-U6-ArpC3 sg1-hSynI-Cre	2/9	pAAV-U6-ArpC3 sg1-hSynI-Cre	Soderling Lab	Figure S3
AAV-U6-ArpC3 sg2-hSynI-Cre	2/9	pAAV-U6-ArpC3 sg2-hSynI-Cre	Soderling Lab	Figure S3
AAV-U6-CARMIL3 sg3-hSynI-Cre	2/9	pAAV-U6-CARMIL3 sg3-hSynI-Cre	Soderling Lab	Figure S7

Supplementary References

1 Bayes, A. *et al.* Comparative study of human and mouse postsynaptic proteomes finds high compositional conservation and abundance differences for key synaptic proteins. *PloS one* **7**, e46683, doi:10.1371/journal.pone.0046683 (2012).

2 Uezu, A. *et al.* Identification of an elaborate complex mediating postsynaptic inhibition. *Science* **353**, 1123-1129, doi:10.1126/science.aag0821 (2016).
