# Supplementary material for: In vivo proximity proteomics of nascent synapses reveals a novel regulator of cytoskeleton-mediated synaptic maturation
Source: Nat Commun. 2019 Jan 23;10:386. doi: 10.1038/s41467-019-08288-w (PMC6344529; doi:10.1038/s41467-019-08288-w)
Supplement: Supplementary file 2 — Reporting Summary [file 41467_2019_8288_MOESM2_ESM.pdf]

Corresponding Author Name: Scott H. Soderling

Manuscript Number: NCOMMS-18-16309B

Reporting Checklist For Nature Communications Life Sciences Articles

This checklist is used to ensure good reporting standards and to improve the reproducibility of published results. For more information, please read Reporting Life Sciences Research.

Figure legends

☑ Check here to confirm that the following information is available in all relevant figure legends (or Methods section if too long):

- the **exact sample size (*n*)** for each experimental group/condition, given as a number, not a range;
- a **description of the sample collection** allowing the reader to understand whether the samples represent **technical or biological replicates** (including how many animals, litters, culture, etc.);
- a **statement of how many times the experiment shown was replicated in the laboratory**;
- **definitions of statistical methods and measures**: (For small sample sizes (n<5) descriptive statistics are not appropriate, instead plot individual data points)
  - very common tests, such as *t*-test, simple χ^2 tests, Wilcoxon and Mann-Whitney tests, can be unambiguously identified by name only, but more complex techniques should be described in the methods section;
  - are tests one-sided or two-sided?
  - are there adjustments for multiple comparisons?
  - **statistical test results**, e.g., ***P* values**;
  - definition of '**center values**' as **median** or **mean**;
  - definition of **error bars** as **s.d.** or **s.e.m.** or **c.i.**

This checklist will not be published. Please ensure that the answers to the following questions are reported in the manuscript itself. We encourage you to include a specific subsection in the Methods section for statistics, reagents and animal models. Below, provide the page number or section and paragraph number (e.g. "Page 5" or "Methods, 'reagents' subsection, paragraph 2").

Statistics and general methods

Statistics and general methods	Reported in section/paragraph or page #:
1. How was the sample size chosen to ensure adequate power to detect a pre-specified effect size? (Give section/paragraph or page #)	Methods (Replicates and Statistics)
For animal studies, include a statement about sample size estimate even if no statistical methods were used.	n/a
2. Describe inclusion/exclusion criteria if samples or animals were excluded from the analysis. Were the criteria pre-established? (Give section/paragraph or page #)	n/a, no data were excluded
3. If a method of randomization was used to determine how samples/ animals were allocated to experimental groups and processed, describe it. (Give section/paragraph or page #)	n/a
For animal studies, include a statement about randomization even if no randomization was used.	n/a
4. If the investigator was blinded to the group allocation during the experiment and/or when assessing the outcome, state the extent of blinding. (Give section/paragraph or page #)	Methods (throughout)
For animal studies, include a statement about blinding even if no blinding was done.	n/a
5. For every figure, are statistical tests justified as appropriate?	Methods (Replication and Statistics)
Do the data meet the assumptions of the tests (e.g., normal distribution)?	Methods (Replication and Statistics)
Is there an estimate of variation within each group of data?	Figure Legends
Is the variance similar between the groups that are being statistically compared? (Give section/paragraph or page #)	Methods (Replication and Statistics)

(Continues on following page)

Reagents

Reported in section/paragraph or page #:

Item	Reported in section/paragraph or page #:
6. To show that antibodies were profiled for use in the system under study (assay and species), provide a citation, catalog number and/or clone number, supplementary information or reference to an antibody validation profile (e.g., Antibodypedia, 1DegreeBio).	Methods (Immunocytochemistry)
7. Cell line identity: a. Are any cell lines used in this paper listed in the database of commonly misidentified cell lines maintained by ICLAC (also available in NCBI Biosample)?	Yes - HEK cells are listed in ICLAC
b. If yes, include in the Methods section a scientific justification of their use – indicate here on which page (or section and paragraph) the justification can be found.	Methods (Cell Lines)
c. For each cell line, include in the Methods section a statement that specifies: - the source of the cell lines	Methods (Cell Lines)
- have the cell lines been authenticated? If so, by which method?	No
- have the cell lines been tested for mycoplasma contamination? In this checklist, indicate on which page (or section and paragraph) the information can be found.	No

Animal Models

Reported in section/paragraph or page #:

Item	Reported in section/paragraph or page #:
8. Report species, strain, sex and age of animals	Methods (Animals)
9. For experiments involving live vertebrates, include a statement of compliance with ethical regulations and identify the committee(s) approving the experiments.	Methods (Animals)

10. We recommend consulting the ARRIVE guidelines (PLoS Biol. 8(6), e1000412,2010) to ensure that other relevant aspects of animal studies are adequately reported.

Human subjects

Reported in section/paragraph or page #:

Item	Reported in section/paragraph or page #:
11. Identify the committee(s) approving the study protocol.	n/a
12. Include a statement confirming that informed consent was obtained from all subjects.	n/a
13. For publication of patient photos, include a statement confirming that consent to publish was obtained.	n/a
14. Report the clinical trial registration number (at ClinicalTrials.gov or equivalent).	n/a
15. For phase II and III randomized controlled trials, please refer to the CONSORT statement and submit the CONSORT checklist with your submission.	n/a
16. For tumor marker prognostic studies, we recommend that you follow the REMARK reporting guidelines.	n/a

(Continues on following page)

Data Availability

Reported in section/paragraph or page #

17. Provide a Data availability statement in the Methods section under "**Data availability**", which should include, where applicable:

- Accession codes for deposited data
- Other unique identifiers (such as DOIs and hyperlinks for any other datasets)
- At a minimum, a statement confirming that all relevant data are available from the authors
- Formal citations of datasets that are assigned DOIs
- A statement regarding data available with restrictions

See our data availability and data citations policy page for more information.

Data deposition in a public repository is mandatory for:

a. Protein, DNA and RNA sequences
b. Macromolecular structures
c. Crystallographic data for small molecules
d. Microarray data

Deposition is strongly recommended for many other datasets for which structured public repositories exist; more details on our data policy are available here. We encourage the provision of other source data in supplementary information or in unstructured repositories such as Figshare and Dryad. We encourage publication of Data Descriptors (see Scientific Data) to maximize data reuse

Response: Methods (Data Availability)

18. If computer code was used to generate results that are central to the paper's conclusions, include a statement in the Methods section under "**Code availability**" to indicate whether and how the code can be accessed. Include version information as necessary and any restrictions on availability.

Response: n/a
